# Regulation of Epidermal Growth Factor Receptor Signaling and Erlotinib Sensitivity in Head and Neck Cancer Cells by miR-7

**DOI:** 10.1371/journal.pone.0047067

**Published:** 2012-10-24

**Authors:** Felicity C. Kalinowski, Keith M. Giles, Patrick A. Candy, Alishum Ali, Clarissa Ganda, Michael R. Epis, Rebecca J. Webster, Peter J. Leedman

**Affiliations:** 1 Laboratory for Cancer Medicine, Western Australian Institute for Medical Research and University of Western Australia Centre for Medical Research, Perth, Western Australia, Australia; 2 School of Medicine and Pharmacology, University of Western Australia, Nedlands, Western Australia, Australia; Northwestern University, United States of America

## Abstract

Elevated expression and activity of the epidermal growth factor receptor (EGFR)/protein kinase B (Akt) signaling pathway is associated with development, progression and treatment resistance of head and neck cancer (HNC). Several studies have demonstrated that microRNA-7 (miR-7) regulates EGFR expression and Akt activity in a range of cancer cell types via its specific interaction with the EGFR mRNA 3′-untranslated region (3′-UTR). In the present study, we found that miR-7 regulated EGFR expression and Akt activity in HNC cell lines, and that this was associated with reduced growth *in vitro* and *in vivo* of cells (HN5) that were sensitive to the EGFR tyrosine kinase inhibitor (TKI) erlotinib (Tarceva). miR-7 acted synergistically with erlotinib to inhibit growth of erlotinib-resistant FaDu cells, an effect associated with increased inhibition of Akt activity. Microarray analysis of HN5 and FaDu cell lines transfected with miR-7 identified a common set of downregulated miR-7 target genes, providing insight into the tumor suppressor function of miR-7. Furthermore, we identified several target miR-7 mRNAs with a putative role in the sensitization of FaDu cells to erlotinib. Together, these data support the coordinate regulation of Akt signaling by miR-7 in HNC cells and suggest the therapeutic potential of miR-7 alone or in combination with EGFR TKIs in this disease.

## Introduction

Head and neck cancer (HNC) is the sixth most common cancer, with approximately 600,000 new cases globally per annum [1]. Despite advances in treatment modalities, the prognosis for many HNC patients that present with advanced or metastatic disease remains poor [2]. The epidermal growth factor receptor (EGFR), a member of the ErbB receptor tyrosine kinase (RTK) family, is overexpressed in greater than 80% of all HNCs and is an independent predictor of poor outcome [3]. EGFR signaling activates a network of downstream pathways, including the phosphoinositide 3-kinase (PI3K)/Akt [4], [5] and Ras/Raf/ERK1/2 [6], [7] pathways, which promote tumor cell proliferation, invasion, metastasis, angiogenesis and apoptosis resistance. Consequently, the EGFR has emerged as a major therapeutic target in HNC.

Cetuximab is a monoclonal antibody (mAb) directed against the EGFR that improved survival of HNC patients treated in combination with radiotherapy or chemotherapy [8], [9]. Small molecule TKIs, such as gefitinib and erlotinib, have also been evaluated in advanced HNC and have had some clinical anti-tumor effect in a small subset of patients [10], [11]. Clinical trials in HNC evaluating the combination of EGFR inhibitors with other therapeutic approaches have also been initiated, as well as studies investigating new generation anti-EGFR agents, such as the anti-EGFR mAb panitumumab and the dual EGFR/HER2 TKI lapatinib [12]. The poor clinical response of HNC to anti-EGFR therapies is due to the inherent and acquired resistance of HNC cells to these agents, which is thought to occur via several mechanisms, including mutations within EGFR and its downstream effectors that activate signaling (eg. loss of PTEN, PI3K mutation or overexpression), compensatory signaling via other RTKs (eg. other ErbB family members, insulin-like growth factor 1 receptor (IGF1R), or c-Met), and the transition from an epithelial to a mesenchymal phenotype (EMT) [12], [13], [14]. Therefore, new approaches are required to effectively inhibit the downstream oncogenic signaling pathways that are activated in an EGFR-independent manner in EGFR inhibitor-resistant HNC.

microRNAs (miRNAs) are short, endogenous, non-coding RNAs that bind to the 3′-untranslated region (3′-UTR) of specific target mRNAs to repress gene expression, either via inducing translational repression or transcript degradation [15]. miRNAs may regulate many gene targets, often in a coordinated fashion within a cellular pathway or network [16], and by doing so they control diverse cellular processes, including development, differentiation, apoptosis and cell cycle progression [17]. Aberrant miRNA expression is a hallmark of many cancers, including HNC, and these miRNAs may act as tumor suppressors or oncogenes [18]. For example, let-7d expression is reduced in many HNCs, causing elevated RAS expression, increased tumor growth and reduced patient survival [19]. In contrast, miR-184 expression is upregulated in tongue squamous cell carcinoma, leading to increased expression of the *c-Myc* oncogene, increased cell proliferation and tumor growth [20]. Recent reports suggest that miRNAs may have utility as novel cancer therapeutics or diagnostic/prognostic biomarkers [18], [21], [22].

We and others have demonstrated that miR-7 inhibits EGFR expression and downstream Akt and ERK1/2 activity in lung, breast, prostate cancer and glioblastoma [23], [24], leading to reduced cell proliferation and survival. The regulation of EGFR expression involves the direct, specific interaction of miR-7 with two miR-7 target sites within the EGFR mRNA 3′-UTR. Furthermore, miR-7 regulates expression of other molecules downstream of EGFR in a coordinate fashion, including RAF1, IRS1, IRS2, PAK1 [23], [24], [25], supporting a tumor suppressor function for miR-7 in these and other cancers. In the present study, we investigated the function of miR-7 on EGFR signaling and growth in HNC *in vitro* and *in vivo*, focusing on cell lines that are sensitive or resistant to the EGFR TKI erlotinib. We hypothesized that miR-7 would inhibit EGFR expression and downstream signaling, and evaluated the synergy between erlotinib and miR-7 in erlotinib-resistant HNC cells. Finally, we performed microarray analyses of two HNC cell lines to identify novel miR-7 targets that would elucidate the molecular mechanisms underlying its tumor suppressor action in HNC. Our findings have broad implications for the treatment of HNC with EGFR-targeted therapies and the use of miR-7 as a novel anti-HNC therapeutic.

## Results

### Differential Sensitivity of HNC Cell Lines to the EGFR Inhibitor Erlotinib

**Figure 1:**
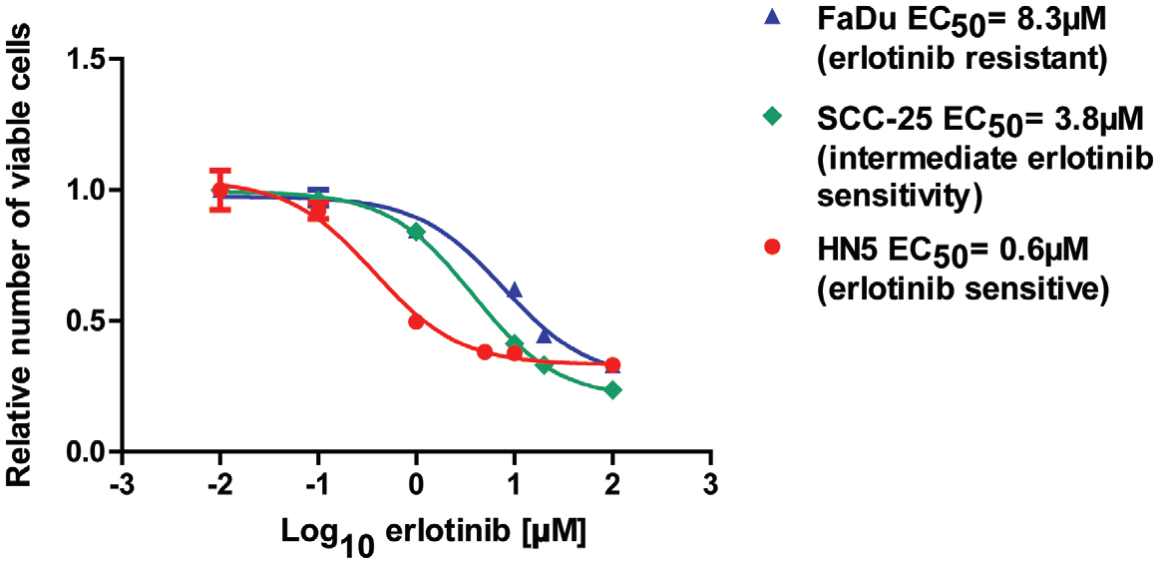
Characterization of erlotinib sensitivity of HN5, FaDu and SCC-25 HNC cells. HN5 (red), FaDu (blue) and SCC-25 (green) cells were seeded in 96-well plates and treated with the EGFR inhibitor erlotinib (final concentration 0–100 µM). The half maximal effective concentration (EC_50_) of erlotinib was determined for each cell line after measurement of the relative number of viable cells by cell titre assay 3 d after the addition of erlotinib. Data are normalized to the lowest concentration of erlotinib. Error bars represent standard deviations. Data are representative of three independent experiments.

To establish the relative sensitivity of HN5, FaDu, and SCC-25 HNC cells to the EGFR inhibitor erlotinib, we performed cell titre assays of cells treated with a broad range of concentrations (Fig. 1). Whereas HN5 cells were highly sensitive to erlotinib (EC_50_ = 0.6 µM), FaDu cells were resistant to the drug (EC_50_ = 8.3 µM). SCC-25 cells displayed intermediate sensitivity to erlotinib (EC_50_ = 3.8 µM) HN5 cells have been reported to overexpress EGFR via a gene amplification [26], and be highly sensitive to EGFR inhibition *in vitro* and *in vivo* [27]. FaDu and SCC-25 cells exhibit moderate EGFR expression and have been shown to exhibit resistance to the EGFR inhibitor gefitinib [28]. Our results are consistent with these reports, whereby FaDu cells are ∼10-fold more resistant to erlotinib than HN5 cells. Taken together, HN5 cells are representative of EGFR inhibitor-sensitive HNC, FaDu cells represent HNC that is resistant to EGFR inhibition, and SCC-25 cells represent HNC with intermediate EGFR-inhibitor resistance. Thus, we can investigate the tumor suppressor activity of miR-7 in each of these settings.

### miR-7 Regulates EGFR Expression and Akt Activity in HNC Cell Lines

**Figure 2:**
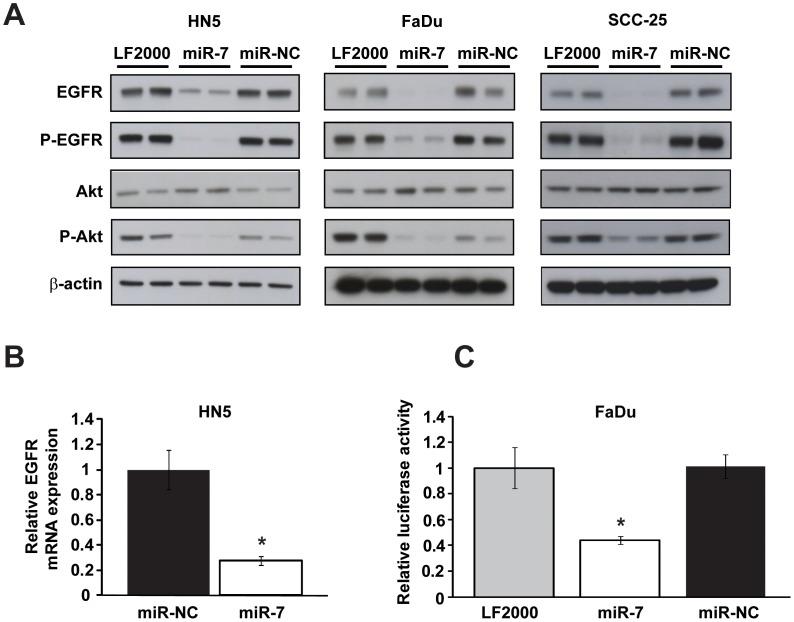
miR-7 regulates EGFR expression and Akt signaling in HNC cell lines. (A) Western blotting analysis of EGFR, P-EGFR, Akt and P-Akt levels in HN5 (left panel) and FaDu (center panel) and SCC-25 (right panel) cells 3 d after transfection with miR-7 or miR-NC precursor molecules or vehicle (LF2000) only. β-actin is included as a loading control. (B) RT-qPCR analysis of EGFR mRNA expression in HN5 cells 24 h after transfection with miR-7 or miR-NC precursor molecules. Data was normalized to GAPDH mRNA expression and expressed relative to miR-NC-transfected cells. (C) Luciferase reporter assay with FaDu cells co-transfected with miR-7 or miR-NC precursor molecules, a firefly luciferase full-length EGFR mRNA 3′-UTR reporter plasmid, and a *Renilla* luciferase reporter plasmid. Firefly luciferase activity was assessed 24 h post-transfection, normalized to *Renilla* luciferase measurements, and data was expressed relative to vehicle (LF2000) only-transfected cells. Error bars represent standard deviations. All data are representative of three independent experiments. *, p<0.001, miR-7 vs miR-NC.

Previously, we and others identified the EGFR mRNA 3′-UTR as a specific target of miR-7 in multiple different cancer cell lines, and demonstrated that miR-7 is a potent inhibitor of EGFR signaling and cell viability [23], [24]. Interestingly, miR-7 attenuated EGFR signaling in EGFR inhibitor-resistant U87MG (glioblastoma) and A549 (non-small cell lung cancer) cells [23], [24]. Given the important role of EGFR signaling in HNC tumorigenesis and treatment, we investigated the capacity of miR-7 to inhibit EGFR expression and downstream signaling in HNC cell lines. We transfected HN5, FaDu and SCC-25 cell lines with miR-7 or a negative control miRNA, miR-NC, and performed western blotting to determine the impact of miR-7 on EGFR expression and Akt activity. miR-7 inhibited EGFR expression and activity (P-EGFR), as well as activity of Akt (P-Akt), a key downstream effecter of EGFR (Fig. 2A). The reduction in Akt activity caused by miR-7 was associated with an increase in the relative levels of total Akt (Fig. 2A), an effect that possibly reflects a feedback mechanism controlling Akt expression in head and neck cancer cells. We did not observe inhibition of ERK1/2 signaling in head and neck cancer cell lines by miR-7 (data not shown). Reverse transcriptase quantitative polymerase chain reaction (RT-qPCR) analysis of HNC cells transfected with miR-7 demonstrated reduced EGFR mRNA expression (HN5 cells, Fig. 2B; FaDu cells, data not shown), a finding consistent with our previous studies with miR-7 in glioblastoma, lung, breast and prostate cancer cells [24]. The reduction in EGFR mRNA by miR-7 is also supported by a recent report suggesting that most miRNAs repress gene expression by promoting mRNA decay [29]. Finally, we confirmed the direct inhibition of EGFR expression by miR-7 via its action upon the EGFR 3′-UTR using reporter gene assays in HNC cells. Co-transfection of HNC cells with miR-7 and a firefly luciferase reporter construct containing the full length EGFR mRNA 3′-UTR yielded significantly lower reporter activity than with miR-NC (FaDu cells, Fig. 2C; HN5 cells, data not shown), confirming that miR-7 interacts with specific EGFR 3′-UTR target sites [24] in HNC cells. Taken together, these results indicate that miR-7 inhibits EGFR mRNA and protein expression and downstream signaling at least in part via direct targeting of the EGFR mRNA 3′-UTR.

### miR-7 Inhibits the Growth of Erlotinib-sensitive HN5 Cells *in vitro* and *in vivo*

**Figure 3:**
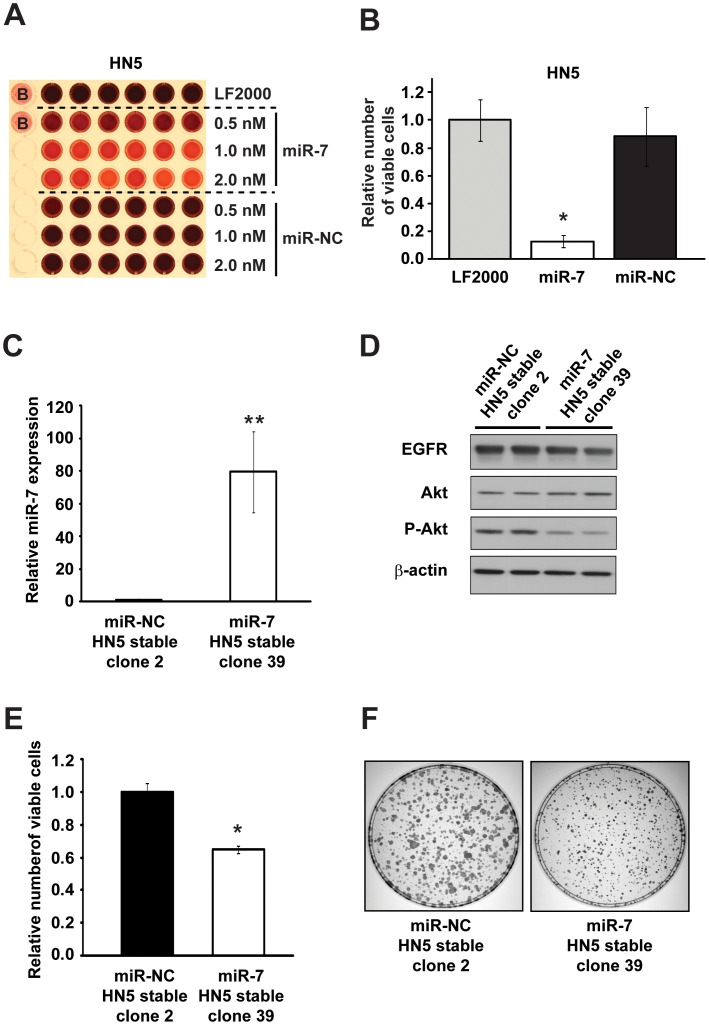
miR-7 inhibits HNC cell growth *in vitro*. (A) Colorimetric cell titre assay of HN5 cells 5 d after transfection in 96-well plates with miR-7 or miR-NC precursor molecules, or vehicle (LF2000) only. (B) Graphical representation of cell titre assay from (A). Data represents the relative number of viable HN5 cells normalized to LF2000-treated HN5 cells. (C) TaqMan RT-qPCR analysis of miR-7 expression in HN5 clones with stable expression of miR-7 (clone 39) or miR-NC (clone 2). Data was normalized to U44 snRNA expression and expressed relative to HN5 miR-NC clone 2. (D) Western blotting analysis of EGFR, Akt and P-Akt levels in HN5 clones with stable expression of miR-7 (clone 39) or miR-NC (clone 2). β-actin is included as a loading control. (E) Manual cell counting of HN5 cells with stable expression of miR-7 (clone 39) or miR-NC (clone 2). Data is expressed relative to miR-NC. (F) Clonogenicity assay of HN5 cells with stable expression of miR-7 (clone 39) or miR-NC (clone 2) 10 d after cells were seeded in 10 cm dishes. Error bars represent standard deviations. All data are representative of three independent experiments. *, p<0.01, miR-7 vs miR-NC; **, p<0.005, miR-7 vs miR-NC.

HN5 HNC cells are highly sensitive to erlotinib and thus represent an excellent model to assess the functional significance of EGFR inhibition by miR-7 *in vitro* and *in vivo*. We investigated the effect of miR-7 on erlotinib-sensitive HN5 cells by measuring cell viability with cell titre assays 5 d after transient transfection of miR-7. Compared to vehicle (LF2000) and miR-NC, miR-7 significantly reduced HN5 cell growth (Fig. 3A and Fig. 3B).

To assess the effect of miR-7 on HN5 tumor growth *in vivo*, we used lentiviral delivery to generate HN5 cells with stable overexpression of miR-7 or miR-NC. Using TaqMan miRNA RT-qPCR we observed ∼80 fold upregulation of miR-7 in HN5 cells with stable miR-7 expression (HN5 stable clone 39), compared to HN5 cells with stable miR-NC expression (HN5 stable clone 2) (Fig. 3C). Analysis of EGFR expression and signaling by western blotting revealed a modest reduction (∼15%; *see Methods 2.7*) in EGFR protein levels in HNC with miR-7 stable expression (Fig. 3D), but a larger (∼25%) reduction in the levels of Akt phosphorylated at serine-473 (P-Akt). We screened multiple HN5/miR-7 clones and observed miR-7 overexpression and similar levels of EGFR and P-Akt in each clone (Fig. S1). Taken together, these findings suggest that the decrease in Akt activity mediated by miR-7 is not merely due to inhibition of EGFR expression and instead is likely to result from a coordinate reduction in expression and activity of multiple molecules responsible for Akt activity.

To determine whether stable miR-7 expression inhibits HN5 growth *in vitro*, we performed manual cell counting of miR-NC HN5 stable clone 2 cells and miR-7 HN5 stable clone 39 cells. These experiments revealed a significant reduction in proliferation of miR-7 stable HN5 cells compared with miR-NC stable HN5 cells (Fig. 3E). To confirm this finding, we assessed the clonogenicity of our HN5 stable cell lines. Stable expression of miR-7 significantly reduced the number and size of colonies formed after 10 d compared with HN5 cells with stable miR-NC expression (Fig. 3F). Together, these results are consistent with those we obtained with transient miR-7 transfection and indicate that the ∼80 fold increase in miR-7 expression in HN5 miR-7 stable clone 39 leads to decreased Akt activity and significantly reduces the *in vitro* growth of HN5 HNC cells.

**Figure 4:**
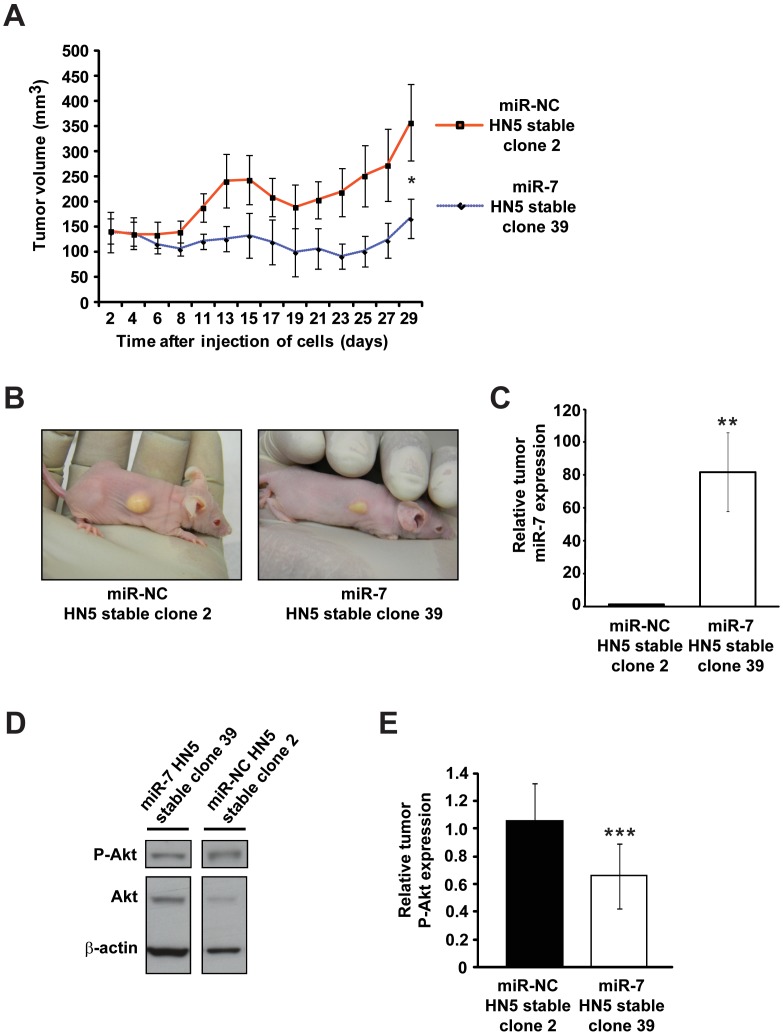
miR-7 inhibits HNC xenograft growth *in vivo*. (A) HN5 tumor xenograft growth following subcutaneous injection of stable HN5 miR-NC clone 2 (red) or miR-7 clone 39 (blue) cells into nude mice. Mean tumor volume (mm^3^) is plotted over time (d). (B) Representative photographs of tumor xenografts for cells with stable miR-NC expression (clone 2, left) and stable miR-7 expression (clone 39, right). (C) TaqMan RT-qPCR analysis of miR-7 expression in HN5/miR-7 and HN5/miR-NC stable tumor xenografts at experimental endpoint. Data was normalized to U44 snRNA expression and miR-7 levels are shown relative to HN5/miR-NC clone 2 tumors. (D) Western blotting analysis of Akt and P-Akt levels between HN5/miR-7 and HN5/miR-NC stable tumor xenografts. β-actin and Akt are included as loading controls. (E) Densitometry analysis of P-Akt levels between HN5/miR-7 (clone 39) and HN5/miR-NC (clone 2) tumor xenografts by western blotting. P-Akt levels were normalized to total Akt expression. Error bars represent standard deviations. *, p<8.0×10^−5^, miR-7 vs miR-NC; **, p<1.0×10^−8^, miR-7 vs miR-NC; ***, p = 0.06, miR-7 vs miR-NC.

We next sought to investigate the impact of stable miR-7 expression on the *in vivo* growth of HN5 tumor xenografts in nude mice. Following subcutaneous injection of miR-7 HN5 stable clone 39 or miR-NC HN5 stable clone 2 cells in the flank of female nude mice, tumor volumes were monitored over a 29 day period. A significant reduction in tumor growth was observed for HN5 xenografts with stable miR-7 expression compared to miR-NC stable HN5 xenografts (Fig. 4A and Fig. 4B). We used TaqMan miRNA qRT-PCR analysis of miR-7 levels to confirm that miR-7 overexpression persisted in the HN5/miR-7 xenografts for the duration of our study (Fig. 4C), and we also observed a reduction in P-Akt levels in the HN5/miR-7 clone 39 xenografts compared with HN5/miR-NC clone 2 xenografts by western blotting (Fig. 4D and Fig. 4E). This is the first demonstration that miR-7 can reduce HNC tumor growth *in vivo*, and is consistent with other recent studies demonstrating that miR-7 reduces the growth of liver cancer, lung cancer and schwannoma xenografts in mice [30], [31], [32]. To confirm our finding and to exclude the possibility of a clonal effect, we performed a tumor formation assay in which HN5 cells were transiently transfected in culture with miR-7 or miR-NC, and after 24 h injected subcutaneously into the flank of female nude mice and tumor formation assessed. Analysis of tumor volumes at 10 d revealed a significant inhibition of tumor formation in mice injected with HN5 cells transiently transfected with miR-7 (HN5/miR-7), compared to mice injected with vehicle-treated or miR-NC-transfected HN5 cells (HN5/miR-NC) (Fig. S2). These data support our observation that stable miR-7 expression inhibits the growth of HN5 tumor xenografts (Fig. 4A and Fig. 4B) and the hypothesis that miR-7 is a potent inhibitor of EGFR signaling, Akt activity, and tumorigenicity in HNC.

### Syngergistic Action of miR-7 and Erlotinib in FaDu and SCC-25 HNC Cells *in vitro*

**Figure 5:**
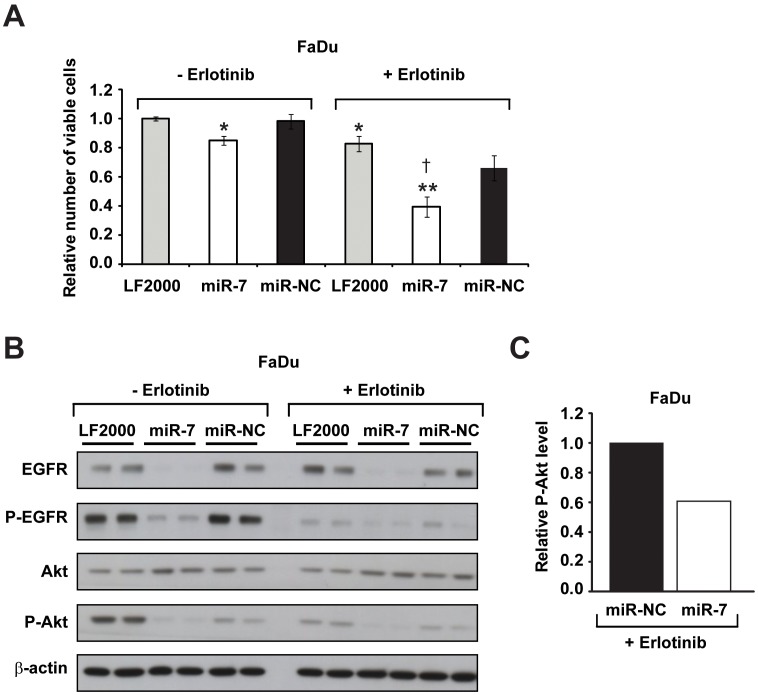
Synergistic inhibition of cell growth and EGFR/Akt signaling in erlotinib-resistant FaDu cells by miR-7 and erlotinib. (A) Cell titre analysis of FaDu cells that were transfected with vehicle only (LF2000), miR-7, or miR-NC for 3 d, and then treated with erlotinib (7.5 µM) or vehicle (DMSO) for a further 4 d. Data is expressed relative to vehicle-transfected, vehicle-treated FaDu cells (LF2000 minus erlotinib, first column). (B) Western blotting analysis of EGFR, P-EGFR, Akt and P-Akt levels in FaDu cells that were transfected with vehicle only (LF2000), or miR-7 or miR-NC for 3 d and then treated ± erlotinib (7.5 µM) for 24 h. β-actin is included as a loading control. (C) Densitometry analysis of P-Akt levels from western blotting between FaDu cells transfected with miR-NC or miR-7 and then treated with erlotinib (7.5 µM) for 24 h. Data is shown relative to miR-NC-transfected cells. Error bars represent standard deviations. All data are representative of three independent experiments. *, p<0.05, miR-7 minus erlotinib vs miR-NC minus erlotinib, and LF2000 plus erlotinib vs LF2000 minus erlotinib; **, p<0.01, miR-7 plus erlotinib vs miR-NC plus erlotinib. † indicates synergy between miR-7 and erlotinib as defined by the Bliss additivism model [34].

As miR-7 can inhibit EGFR expression as well as activity of Akt, we hypothesized that it might enhance the efficacy of anti-EGFR therapeutics with HNC cells; that is, the combination of miR-7 and erlotinib might synergistically inhibit the growth of HNC cells. To investigate this, we utilized FaDu cells that are resistant to erlotinib (EC_50_ = 8.3 µM; ∼10 fold higher than HN5 cells). FaDu cells were transiently transfected with miR-7, miR-NC, or vehicle only, and treated at 3 d post-transfection with a sub-EC_50_ and clinically-relevant [33] dose (7.5 µM) of erlotinib (or vehicle). After a further 4 d, cell viability was assessed by cell titre assay (Fig. 5A). miR-7 alone (without erlotinib) and erlotinib alone (vehicle only; LF2000) each produced a small yet statistically significant inhibition of FaDu cell growth. However, the combination of miR-7 and erlotinib produced an even larger reduction in cell growth, an effect that was determined by the Bliss additivism model [34] to be synergistic, where E_Bliss_ = E_miR-7_+E_erlotinib_–E_miR-7_×E_erlotinib_ = 0.15+0.18–0.15×0.18 = 0.30, and the experimentally observed fractional inhibition (E_observed_) with miR-7 plus erlotinib was 0.61. We also observed synergy between miR-7 and erlotinib in SCC-25 HNC cells (Fig. S3), where E_Bliss_ = E_miR-7_+E_erlotinib_−E_miR-7_×E_erlotinib_ = 0.61+0.35−0.6×0.35 = 0.75, and the experimentally observed fractional inhibition (E_observed_) with miR-7 plus erlotinib was 0.84.

In parallel studies, we used western blotting to assess the impact of miR-7, erlotinib, and the combination of miR-7 and erlotinib on EGFR expression/activity and the activity of Akt in FaDu cells (Fig. 5B). We observed inhibition of EGFR phosphorylation (P-EGFR) with both erlotinib and miR-7, while miR-7 also reduced basal EGFR expression. Importantly, the combination of miR-7 and erlotinib produced a greater reduction in P-EGFR expression than was observed with either erlotinib or miR-7 alone, and densitometry confirmed that P-Akt levels were lower with the combination of miR-7 and erlotinib than with miR-NC and erlotinib (*see Methods 2.7*; Fig. 5C). Taken together, these data indicate that the combination of miR-7 and erlotinib works synergistically in HNC cells, to simultaneously inhibit EGFR expression and activity, Akt activity, and cell growth.

### Microarray Analysis of miR-7-regulated Genes in HN5 and FaDu HNC Cells

**Figure 6:**
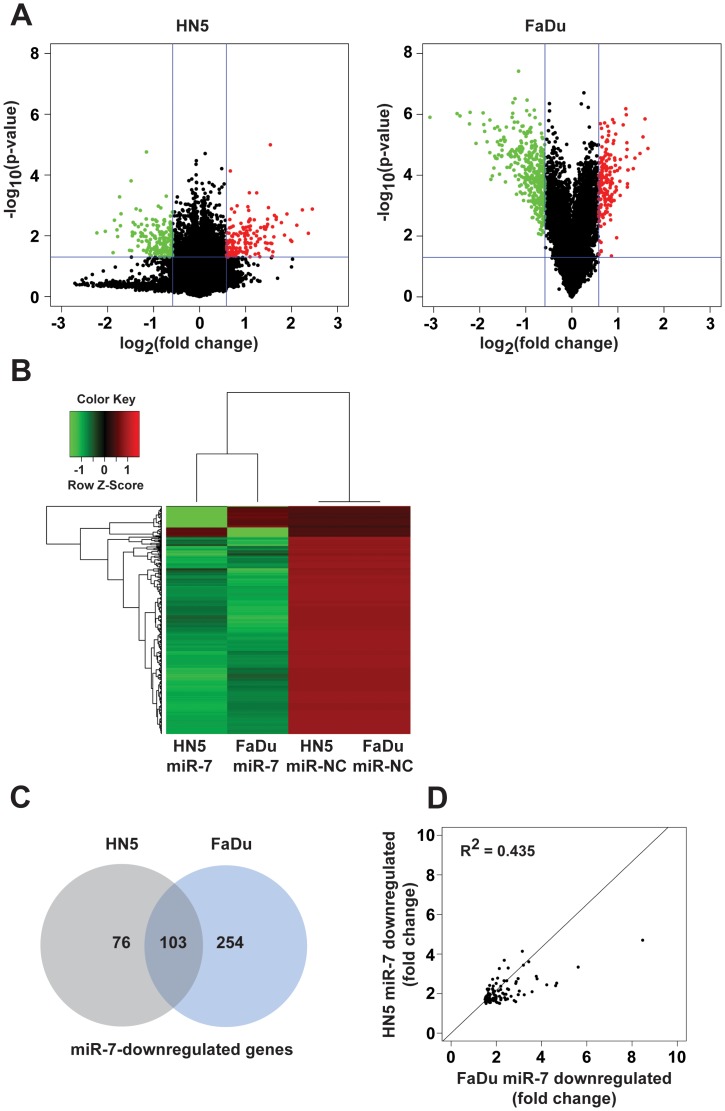
Microarray analysis of miR-7-downregulated genes in HN5 and FaDu cells. (A) Volcano plots representing array probes between HN5 (left) and FaDu (right) cells 24 h after transfection with miR-7 or miR-NC precursor molecules. Assigning a cut off of ±1.5-fold change (miR-7 vs miR-NC) and p<0.05, significantly downregulated probes are in green and significantly upregulated probes are in red. (B) Cluster analysis of miR-7-downregulated genes in HN5 and FaDu cells, where green and red shading corresponds to downregulated and upregulated genes, respectively. (C) Venn diagram of miR-7-downregulated genes in HN5 and FaDu cells. (D) Scatter plot of miR-7-downregulated genes common to HN5 and FaDu cells (R^2^ = 0.435, p<0.001).

To gain further insight into the mechanism of action of miR-7 in both HN5 and FaDu cells, we performed microarray analyses in parallel of total RNA isolated from HN5 and FaDu cells that were transfected with miR-7 or miR-NC. We elected to harvest RNA for microarray analysis at 24 h post-transfection with miRNA, to maximise specificity and sensitivity, based on our previous experience with miR-7 transfection of A549 cells [24] and a report suggesting that downregulation of indirect miR-124 target mRNAs in HepG2 liver cancer cells commenced at ∼32 h after transfection [35]. After data normalization, we reasoned that direct miR-7 target genes in HN5 and FaDu cells would be significantly downregulated following miR-7 transfection. We refined these gene lists by assigning a cut off for downregulation of at least −1.5 fold and with a significance of p<0.05 (Fig. 6A), and performed cluster analysis of the resulting genes that were significantly downregulated by miR-7 relative to miR-NC in HN5 or FaDu cells (Fig. 6B). This revealed overlap in the genes downregulated by miR-7 between the two cell lines. One hundred and seventy nine mRNAs were significantly downregulated by miR-7 in HN5 cells (Table S1), 357 mRNAs were significantly downregulated in FaDu cells by miR-7 (Table S2), and 103 mRNAs were commonly downregulated in both HN5 and FaDu cells (Table S3). A Venn diagram consisting of the miR-7 downregulated genes for each cell line (Fig. 6C) highlights the intersection of miR-7 downregulated mRNAs between HN5 and FaDu cells, which represents a miR-7 target signature in HNC cells. Scatter plot analysis indicated a strong correlation (R^2^ = 0.435, p<0.001) between the fold-decrease in expression of the 103 genes in response to miR-7 between HN5 and FaDu cells (Fig. 6D). We also used RT-qPCR analysis to confirm the downregulation of RAF1 and transforming growth factor alpha (TGFA) mRNA by miR-7 in our microarray analyses of both HN5 and FaDu cells (Fig. S4). These observations suggest miR-7 has the capacity to target the EGFR, its downstream pathways and also an EGFR ligand, such as TGFA.

To validate our approach of combining the miR-7 downregulated gene sets for HN5 and FaDu cells to identify common target mRNAs, we used Ingenuity Pathway Analysis (IPA) to assess the proportion of genes that were significantly downregulated by miR-7 and were moderate or high-confidence predicted, or proven, miR-7 targets. IPA identified 91/179 (54%) downregulated HN5 mRNAs as being predicted or proven miR-7 targets, 113/357 (32%) downregulated FaDu mRNAs as being predicted or proven miR-7 targets, and 57/103 (55%) downregulated mRNAs common to both HN5 and FaDu cells as being putative or actual miR-7 targets, emphasizing the enrichment of the combined miR-7 downregulated gene set with putative or validated miR-7 target mRNAs and suggesting that it represents a miR-7 target signature in HNC cells.

**Figure 7:**
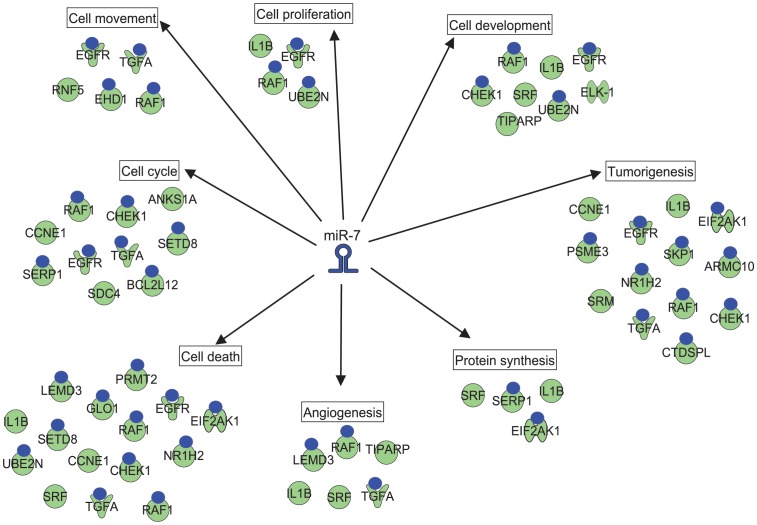
A functional miR-7 target signature in HNC cells. miR-7-downregulated genes common to both HN5 and FaDu cells (103) were assigned to annotated cancer-associated processes using IPA software. These included “cell cycle”, “cell movement”, “cell proliferation”, “cell development”, “tumorigenesis”, “protein synthesis”, “angiogenesis” and “cell death”. Official gene symbols are used for each miR-7-downregulated gene and a blue circle indicates that a gene is a predicted or validated target of miR-7 by IPA analysis.

To identify the functional significance of the miR-7 target gene set common to HN5 and FaDu cells, we used IPA to assign functions to the 103 genes that were downregulated by miR-7 in both cell lines. A subset of the 103 genes - of which more than half were predicted or validated miR-7 targets - was associated with functions that represent a broad range of processes involved in tumor growth and progression, including “cell proliferation”, “cell development”, “tumorigenesis”, “protein synthesis”, “angiogenesis”, “cell death”, “cell cycle”, and “cell movement” (Fig. 7). This finding highlights the capacity of miR-7 to co-ordinately regulate multiple oncogenic processes in cancer cells.

**Figure 8:**
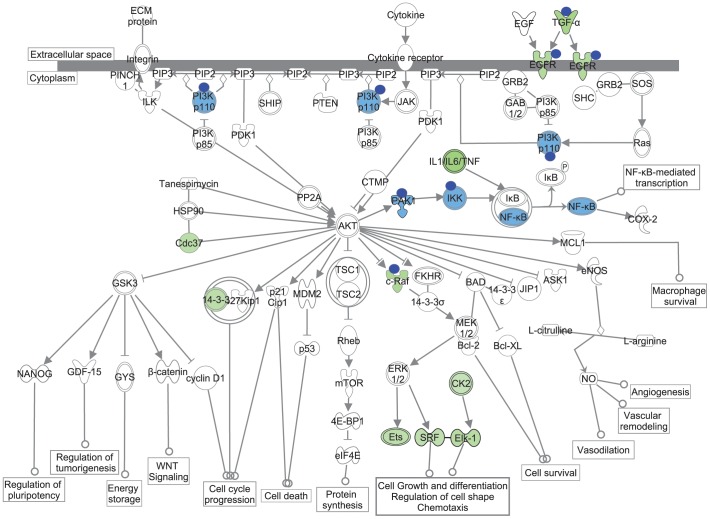
Coordinate regulation of EGFR/Akt signaling by miR-7 in HNC cells. Schematic representation of molecules in the EGFR/Akt signaling pathway that are inhibited by miR-7 in HNC. IPA software was used to map common miR-7-downregulated genes (shown in green) in FaDu and HN5 cells onto the canonical PI3K/Akt pathway. The density of shading represents the fold-change downregulation of a gene by miR-7. Blue circles indicate that a gene is a predicted or validated target of miR-7 by IPA analysis. Several genes belonging to the PI3K/Akt pathway that were downregulated by miR-7 in HN5 cells only are shaded in light blue.

To gain further insight into the function of miR-7 as a regulator of EGFR signaling and Akt activity – with the capacity to sensitize HNC cells to erlotinib - we used IPA to assign the group of genes downregulated by miR-7 in HN5 cells and FaDu cells to the canonical “PI3K/Akt signaling” pathway (Fig. 8). While PIK3CD, PAK1, IKK and NF-κB were downregulated by miR-7 in HN5 but not FaDu cells, the majority of miR-7 downregulated genes associated with the PI3K/Akt pathway were decreased in both HN5 and FaDu cells, suggesting that they represent a definitive miR-7 target signature associated with PI3K/Akt signaling downstream of EGFR. We hypothesize that the synergy between miR-7 and erlotinib is at least in part due to a miR-7-mediated downregulation of multiple molecules associated with Akt activity in HNC cells.

## Discussion

In the present study, we demonstrated that miR-7 regulates EGFR expression and downstream signaling in HN5 and FaDu HNC cell lines that have differential sensitivity to the EGFR TKI erlotinib. We found that miR-7 inhibits growth of erlotinib-sensitive HN5 cells *in vitro* and *in vivo*, and that miR-7 can sensitize erlotinib-resistant FaDu cells to erlotinib. Microarray analysis of miR-7-transfected HN5 and FaDu cells identified a common target gene signature for miR-7, providing insight into its tumor suppressor activity in the context of several cancer-associated functions, such as cell proliferation and cell movement. Finally, analysis of our microarray data suggested that miR-7 sensitizes EGFR-inhibitor resistant HNC cells to erlotinib by coordinately regulating expression of multiple molecules associated with activity of Akt, a critical downstream effector of EGFR and an emerging therapeutic target in cancer.

A key finding of our study is that miR-7 has the capacity to sensitize erlotinib-resistant HNC cells to erlotinib, with the combination of erlotinib and miR-7 exhibiting a synergistic decrease in cell proliferation compared to erlotinib or miR-7 alone. This observation is important given that resistance of HNC to EGFR inhibitors is common and a major clinical problem [12]. Our data also build on a recent report by Rai and co-workers in which delivery of miR-7 to EGFR inhibitor-resistant lung cancer cells suppressed growth *in vitro* and *in vivo*, an effect that was associated with decreased expression of EGFR as well as its downstream kinases RAF1 and IRS1 [31]. Newer targeted cancer therapies often inhibit multiple kinases to overcome compensatory signaling and tumor resistance. For instance, in preclinical HNC models, inhibition of c-Met, an oncogenic RTK, synergizes with erlotinib [36].

In an attempt to identify mechanisms underlying the synergy between miR-7 and erlotinib, our microarray analysis of FaDu cells identified a number of putative or validated miR-7 target molecules that are associated with Akt signaling and implicated in EGFR inhibitor resistance (Fig. 8). In addition to EGFR and RAF1, which are validated direct target mRNAs of miR-7 [24], we also identified the growth factor and EGFR ligand TGFA as a putative miR-7 target in HNC cells. TargetScan (v6.1) analysis [37] indicates that there are five poorly-conserved, predicted miR-7 target sites within the >3 kb TGFA mRNA 3′-UTR, suggesting that miR-7 could directly regulate TGFA expression. Interestingly, a study by Hickinson and co-workers identified TGFA and EGFR as belonging to a set of biomarkers that predict for *in vitro* gefitinib sensitivity of HNC cell lines [38]. Our observation that miR-7 is capable of regulating three key aspects of the EGFR signaling pathway (the ligand, the receptor and multiple molecules downstream of the receptor) illustrates how a single miRNA can orchestrate coordinated inhibition of a major signaling pathway at multiple levels. We are unaware of any other miRNA to date that has such breadth of action on a single signaling pathway.

Downregulation of interleukin-1β (IL1B) by miR-7 may also contribute to the sensitization of FaDu cells to erlotinib. IL1B promotes the shedding of heparin-binding EGF-like growth factor (HB-EGF), an EGFR ligand, in gastric cancer cells [39]. Thus, the paracrine and autocrine action of TGFA and HB-EGF to promote HNC tumorigenesis [40] may be inhibited by miR-7, thereby augmenting the activity of erlotinib. Of interest, Kong and co-workers suggest that the decreased expression of miR-7 in gastric cancer is associated with elevated expression of IL1B and an inflammatory response [41]. Our microarray analyses indicated that IL1B was downregulated following transfection of both HN5 and FaDu cells with miR-7, consistent with a potential regulatory pathway existing between these molecules in cancer. Thus, our data suggest miR-7 has the capacity to inhibit EGFR and its ligands TGFA and HB-EGF, possibly via decreasing IL1B expression.

The downregulation of CK2 and Cdc37 by miR-7 is another potential mechanism responsible for the sensitization of FaDu cells to erlotinib (Fig. 8). Binding and phosphorylation of Cdc37 by the protein kinase CK2 allows it to recruit Hsp90 and to phosphorylate other kinases, including Akt and RAF1 [42]. CK2A also phosphorylates and activates Akt directly [43]. A recent study demonstrated that treatment of lung cancer or squamous cell carcinoma cells with a combination of erlotinib and the novel CK2 inhibitor CX-4945 led to enhanced inhibition of PI3K/Akt signaling and increased anti-tumor efficacy *in vitro* and *in vivo* [44]. These data suggest that inhibition of CK2 and Cdc37 expression by miR-7 may reduce Akt activity and enhance the sensitivity of HNC cells to erlotinib. We also observed inhibition of the E twenty-six (ETS)-like transcription factor 1 (ELK1) expression by miR-7 in FaDu cells. ELK1 is a convergence point for signaling pathways downstream of EGFR, including PI3K/Akt, and RNAi knockdown of ELK1 reduces the growth and survival of U138 glioblastoma cells [45]. Similarly, the ELK1 cofactor, serum response factor (SRF), was downregulated in FaDu cells by miR-7. Overexpression of SRF promotes the growth and migration of liver cancer cell lines and promotes a mesenchymal phenotype [46], a feature that has been associated with the resistance of cancer cells to EGFR inhibitors [47]. While this manuscript was in preparation, a publication by Kong and co-workers [48] reported that miR-7 inhibits EMT in breast cancer cells, at least in part by regulating expression of focal adhesion kinase (FAK). We found that FaDu cells transfected with miR-7 exhibited increased protein expression of the epithelial marker E-cadherin (Fig. S5), suggesting that miR-7 may sensitize HNC cells to erlotinib in part by reversing the EMT process.

Our approach of combining miR-7-downregulated gene sets from both HN5 and FaDu HNC cells aimed to increase the specificity of the putative miR-7 target signature we have identified. We observed considerable overlap (103 miR-7-downregulated genes) between HN5 and FaDu cells, and of these, 57 genes (55%) were predicted or validated miR-7 targets. To understand their relevance to HNC biology, we classified them according to several functional processes central to tumorigenesis (Fig. 7), including “cell cycle”, “cell movement”, “cell proliferation”, “cell development”, “tumorigenesis”, “protein synthesis”, “angiogenesis”, and “cell death”. Interestingly, bioinformatic analysis has previously linked miR-7 to the control of cell cycle progression in breast cancer [49]. A majority (18/27; 67%) of the miR-7 downregulated genes associated with these functions were validated or predicted miR-7 targets, suggesting that they represent a common miR-7 target signature that determines its tumor suppressor activity in HNC.

Of relevance to this study, Jiang and co-workers reported IGF1R expression, but not EGFR expression, being inhibited by miR-7 in tongue squamous cell carcinoma cells [50]. In contrast, our work with HN5 tongue carcinoma cells in this paper indicates that miR-7 downregulates EGFR expression at both the mRNA and protein levels (Fig. 2A and Fig. 2B). It is possible that the actual targets of miR-7 may vary between different tumors of the same type, possibly via differences in target mRNA expression and splicing or the expression and activity of RNA-binding proteins that modulate miRNA function [51]. Indeed, we observed unique sets of genes were downregulated by miR-7 in HN5 and FaDu cells (Fig. 6C), where the tumor suppressor effects of miR-7 are likely to result from repression of genes common to both cell lines as well as unique genes that are critical to one cell line or the other. PAK1 is a known miR-7 target in breast [25] and schwannoma [32] tumors. In our study, PAK1 was downregulated by miR-7 in HN5 cells but not in FaDu cells (Fig. 8). Therefore, the role of a miRNA as a therapeutic target or biomarker should be assessed in the context of each tumor and cell type.

A number of recent reports support our conclusion that miR-7 has significant potential as a cancer therapeutic. Fang and co-workers identified PI3K as a target of miR-7 in liver cancer cells, a finding consistent with our own observation in HN5 cells (Fig. 8), and demonstrated that miR-7 could decrease tumor growth and lung metastasis *in vivo* [30]. Lee and co-workers found that miR-7 could increase the sensitivity of squamous cell carcinoma, breast cancer, lung cancer, and glioblastoma cells to radiation [52]. In addition to strategies to deliver synthetic miR-7 systemically or intra-tumorally, small molecules that upregulate miR-7 could be used therapeutically to inhibit EGFR/Akt signaling and reduce tumorigenicity. For instance, inhibition of the ubiquitin-specific peptidase Usp18 has been shown to increase miR-7 expression in several cancer cell lines, decreasing growth and cell survival [53], and thus a small molecule inhibitor of Usp18 may restore the tumor suppressor activity of miR-7 to cancer cells.

In conclusion, our data indicate that miR-7 inhibits EGFR expression and downstream signaling in HNC cells, and that miR-7 has the capacity to inhibit HNC growth *in vitro* and *in vivo* and to sensitize HNC cells to erlotinib. Microarray profiling suggests that a subset of target mRNAs mediate these effects of miR-7 in HNC cells, and that miR-7 is unique in its ability to inhibit EGFR signaling at the level of receptor, its ligand and downstream signaling pathways. These studies suggest that the therapeutic delivery of miR-7 may inhibit tumor growth and metastasis, sensitize tumors to conventional therapies, and improve patient survival.

## Methods

### Cell Lines, Cell Culture, Erlotinib, miRNA Precursors and Plasmid DNA

The HNC cell line HN5 [54] was derived from a tongue carcinoma from a 73 year old male and was kindly provided by A/Prof. Terrance Johns (Monash Institute of Medical Research). The identity of the HN5 cell line was verified by short tandem repeat (STR) profiling at CellBank Australia (Children’s Medical Research Institute, Westmead, Australia). The HNC cell line FaDu [55] was derived from a pharyngeal carcinoma from a 56 year old male and was obtained from the American Type Culture Collection (ATCC). The SCC-25 cell line [56] was derived from a tongue carcinoma from a 70 year old male and was obtained from ATCC. HN5 and FaDu cells were cultured in Dulbecco’s Modified Eagles Medium (DMEM) (Invitrogen) supplemented with 10% fetal bovine serum (FBS) at 37°C in 5% CO_2_. SCC-25 cells were cultured in DMEM/F-12 medium (Invitrogen) supplemented with 400 ng/ml hydrocortisone (Sigma-Aldrich). For all experiments, cell lines were used within 20 passages of initial stock and confirmed to be free of mycoplasma contamination.

Erlotinib (LC Laboratories; Woburn, Massachusetts) was prepared as a 23 mM stock solution in 96% (v/v) dimethyl sulfoxide (DMSO) (Sigma-Aldrich) and 4% (v/v) MilliQ water.

Synthetic miRNA precursor molecules were sourced from Ambion. Molecules corresponding to human miR-7 (Pre-miR miRNA Precursor Product ID: PM10047) and a negative control miRNA (miR-NC; Pre-miR miRNA Precursor Negative Control #1, Product ID: AM17110) were prepared as 50 µM stock solutions in RNase-free water. In experiments testing the effects of miR-7 and/or erlotinib, vehicle control cell cultures were treated with an equivalent v/v dilution of DMSO (in place of erlotinib) or Lipofectamine 2000 (Invitrogen) (in place of miR-7 and miR-NC).

The following DNA plasmids were used: pRL-CMV *Renilla* luciferase reporter (Promega) and pmiR-REPORT-EGFR 3′-UTR firefly luciferase reporter vector [24]. All plasmids were verified by DNA sequencing prior to use.

### miRNA Precursor Transfections

miRNA transfection experiments were performed as described [57]. Briefly, cells were seeded at a density of 4.5×10^5^ (FaDu) or 5.0×10^5^ (HN5 and SCC-25) cells in 6 well plates or seeded at a density of 5.0×10^3^ (FaDu and HN5) in 96 well plates, and transfected using Lipofectamine 2000 with miR-7 or miR-NC precursor molecules at final concentrations ranging from 1–60 nM. Cells were harvested at 24 h for RNA extraction and transient mouse injection or 3 d for protein extraction. In erlotinib experiments, FaDu cells were plated as above and erlotinib (7.5 µM) was added 3 d after transfection for 24 h, after which cells were harvested for protein extraction.

### Cell Viability and Erlotinib Sensitivity Assays

To measure cell viability, FaDu and HN5 cells were seeded in 96 well plates at a density of 5.0×10^3^ cells per well and transfected with miRNA precursor molecules (see 2.2). Cell viability was measured 5 d after transfection using a CellTitre 96 Aqueous One Solution Cell Proliferation Assay Kit (Promega) as per manufacturer’s instructions and a FLUOstar OPTIMA microplate reader (BMG Labtech). The 96 well plates were digitally photographed (Canon EO3 400D). In FaDu and SCC-25 cell transfections where erlotinib was administered following transfection with miRNA precursors, cells were plated as above and erlotinib (FaDu: 7.5 µM; SCC-25∶4 µM) was added 3 d after transfection for 4 d, after which cell viability was measured (i.e. 7 d after transfection).

To measure erlotinib sensitivity, FaDu, SCC-25 and HN5 cell lines were seeded in 96 well plates at a density of 5.0×10^3^ cells per well. Fresh media containing varying concentrations of erlotinib was added 24 h after initial cell plating. Cell viability was measured 3 d after addition of erlotinib using the CellTitre 96 Aqueous One Solution Cell Proliferation Assay Kit (Promega) as per manufacturer’s instructions.

### Generation of Transient and Stable Cell Lines for Xenograft Studies

Transient miR-7 and miR-NC overexpressing HN5 cells (Fig. S2) were generated by transfection with miR-7 or miR-NC precursor molecules as above (section 2.2).

HN5 cells stably expressing miR-7 or miR-NC were generated by transduction using miRIDIAN shMIMIC miR-7 or miR-NC lentiviral particles as per manufacturer’s instructions (Thermo Fisher Scientific). Cells were seeded at a density of 5.0×10^3^ cells in 96 well plates. After 24 h, cells were incubated with viral particles (final multiplicity of infection (MOI) of 5) and polybrene at a final concentration of 5 µg/ml for 18 h. Following this, cells were cultured in complete growth media for 24 h followed by selection of positive clones with 0.5 µg/ml puromycin (Integrated Sciences) and separation of monoclonal populations of positive cells using ring cloning as described [58].

### Reverse Transcription and Quantitative Polymerase Chain Reaction (RT-qPCR)

Total RNA was extracted from cell lines with TRIzol reagent (Invitrogen) and treated with DNase I (Promega) to eliminate contaminating genomic DNA. For RNA extractions from tumor tissue, samples were first homogenized in Trizol reagent by 2×45 sec pulses with 2.8 mm ceramic beads, using a Precellys 24 Homogenizer (Bertin Technologies). For RT-QPCR analysis of EGFR, RAF1, TGFA and glyceraldehyde 3-phosphate dehydrogenase (GAPDH) mRNA expression, 0.5 µg of total RNA was reverse transcribed into cDNA with random hexamers using Thermoscript (Invitrogen). Real-time PCR for EGFR, RAF1, TGFA and GAPDH cDNA was performed on a RotorGene 6000 instrument (Qiagen) using the SensiMix*Plus* SYBR Kit (Bioline, Quantace) and EGFR, RAF1, TGFA and GAPDH primers from PrimerBank [59]: EGFR-F, 5′ -GCG TTC GGC ACG GTG TAT AA- 3′; EGFR-R, 5′ -GGC TTT CGG AGA TGT TGC TTC- 3′; RAF1-F, 5′ -GCA CTG TAG CAC CAA AGT ACC- 3′; RAF1-R, 5′ -CTG GGA CTC CAC TAT CAC CAA TA- 3′; TGFA-F, 5′ -TGT AAT CAC CTG TGC AGC CTT T- 3′; TGFA-R, 5′ -GTG GTC CGC TGA TTT CTT CTC T- 3′; GAPDH-F, 5′ -ATG GGG AAG GTG AAG GTC G- 3′; GAPDH-R, 5′ -GGG GTC ATT GAT GGC AAC ATT A- 3′. Single peak melt curves and reaction efficiencies between 0.9 and 1.1 were achieved. Expression of EGFR, RAF1 and TGFA mRNA relative to GAPDH mRNA was determined using the 2^−ΔΔCT^ method [60].

For analysis of miR-7 expression by RT-qPCR, reverse transcription and PCR were carried out using the TaqMan miRNA assay kit (Applied Biosystems) for hsa-miR-7 (Part #4373014) and U44 snRNA (Part #4427975) with a Rotor-Gene 6000 thermocycler (Qiagen) according to the manufacturer’s instructions. Statistical analyses of RT-qPCR data were performed using GenEx software (MultiD).

### Luciferase Reporter Gene Assays

Luciferase reporter assays were performed as described previously [57]. Cells were seeded at a density of 2.0×10^5^ cells per well in 24 well plates and co-transfected using Lipofectamine 2000 (Invitrogen) with miR-7 or miR-NC precursor molecules (1 nM), and 100 ng per well of firefly luciferase reporter DNA and 5 ng per well of pRL-CMV *Renilla* luciferase reporter as a transfection control. Lysates were collected 24 h after transfection and each supernatant was assayed for firefly and *Renilla* luciferase activity using a Dual-Luciferase Reporter Assay System (Promega) and a FLUOstar OPTIMA luminometer (BMG Labtech). Relative luciferase expression was determined by normalising firefly luciferase values to *Renilla* luciferase values.

### Protein Extraction and Immunoblotting

Cytoplasmic protein extracts were prepared from cell lines as described [57]. Protein was extracted from tumor tissues using a lysis buffer containing 20 mM Tris-Cl pH 7.4, 40 mM KCl and 1% Triton X-100 and homogenization with 2.8 mm ceramic beads, using a Precellys 24 Homogenizer (Bertin Technologies). Proteins were resolved on NuPAGE 4–12% Bis-Tris gels (Invitrogen) and transferred to polyvinylidene difluoride (PVDF) membranes (Roche). Membranes were probed with anti-EGFR rabbit monoclonal antibody (1∶5000, Abcam: ab52894-100), anti-phospho-EGFR (Tyr1173) goat polyclonal antibody (1∶750, Santa Cruz Biotechnology Inc.: sc-12351), anti-Akt rabbit polyclonal antibody (1∶1000, Cell Signaling Technology, Inc.: # 9272), anti-phospho-Akt (Ser473) rabbit monoclonal antibody (1∶500, Cell Signaling Technology, Inc.: # 4060S), anti-E-cadherin rabbit monoclonal antibody (1∶1000, Cell Signaling Technology, Inc.: # 3195S), or anti-β-actin mouse monoclonal antibody (1∶15,000, Abcam: ab6276-100). Secondary horseradish peroxidase linked anti-rabbit-IgG (1∶10,000, GE Healthcare: # NA934V), horseradish peroxidase linked anti-mouse-IgG (1∶10,000, GE Healthcare: # NA931V) and horseradish peroxidase linked anti-goat-IgG antibodies (1∶10,000, Santa Cruz Biotechnology, Inc.: sc-2020) were used prior to detection with an ECL Plus Western Blotting Detection System (GE Healthcare). Proteins of interest were quantitated by obtaining pixel densities of each band and correcting with a background pixel density using Quantity One software (BioRad).

### Clonogenicity Assays and Manual Cell Counting

For clonogenicity assays, HN5 cells stably transfected with miR-7 (clone 39) or miR-NC (clone 2) were seeded at a density of 1.0×10^3^ cells into 100 mm dishes and cultured in DMEM containing 0.5 µg/ml puromycin until visible colonies appeared. Twelve d after plating, cells were fixed by incubating with 100% methanol at -20°C for 10 min. Fixed cells were stained with 0.5% (w/v) crystal violet in 25% methanol. The 100 mm dishes were digitally photographed (Canon EOS 400D) and media containing puromycin was replenished 3 times per week.

For manual cell counting, stable HN5 miR-7 and miR-NC cells were seeded in 12-well plates at a density of 2.5×10^3^ cells per well in quadruplicate and cultured for 7 d. Cells were trypsinized, centrifuged and resuspended in 100 µL DMEM. Viable cells were visualised by trypan blue staining and counted using a Countess Automated Cell Counter (Invitrogen).

### Animals

Animal experiments were conducted in female, 4–6 week old, athymic, BALB/c nu/nu nude mice (Animal Resources Centre; Western Australia). Use of all animals was in accordance with the guidelines of the University of Western Australia Animal Ethics Committee under Ethics Approval numbers RA-3-100-982 (transient mouse injection; Fig. S2) and RA-3-100-942 (lentiviral, stable mouse injection; Fig. 4). All efforts were made to minimise suffering of animals.

### *In vivo* Tumor Xenograft Formation Assays

HN5 cells grown *in vitro* were transfected with either Lipofectamine 2000 only, 60 nM miR-7 or 60 nM miR-NC as above (section 2.2). Twenty four h post-transfection, HN5 cells (2.0×10^6^/mouse) were resuspended 1∶1 in 150 µL of RPMI-1640 (Invitrogen) +10% FBS and Matrigel Matrix High Concentration (BD Bioscience). Cells were subcutaneously injected into the right flank of 20 mice (a randomized group of 4 mice were injected with Lipofectamine 2000 control-treated cells, 8 mice with miR-7-treated cells and 8 mice with miR-NC-treated cells). Once palpable, tumors were measured with digital calipers (Hare & Forbes Machinery House) and tumor volume was calculated using the following formula: (length×width^2^)/2 = tumor volume (mm^3^) [61].

### *In vivo* Tumor Xenograft Growth Assays

Tumor xenograft experiments using HN5 cells stably expressing miR-7 or miR-NC generated as above (lentiviral method, section 2.4) were performed in 20 mice randomized into 2 groups (10 mice per group). Injections consisted of either miR-7 HN5 stable clone 39 or miR-NC HN5 stable clone 2. A total of 2.0×10^6^ cells in 150 µL of a 1∶1 suspension of serum-free RPMI-1640 (Invitrogen) and Matrigel Matrix High Concentration (BD Biosciences) were subcutaneously injected into the right flank of each mouse. Once palpable, tumors were measured as described above (section 2.10).

### cDNA Microarray Expression Profiling

Total RNA was isolated from HN5 and FaDu cells, 24 h after transfection with miR-7 or miR-NC precursor molecules (30 nM) using TRIzol reagent (Invitrogen). The quantity and integrity of extracted RNA was confirmed using a 2100 Bioanalyzer (Agilent Technologies) before samples were deemed suitable for array analysis. Gene expression profiling by microarray hybridization was performed with a minimum of two experimental replicates by the Australian Genome Research Facility (AGRF; Victoria, Australia) using Human-6 v3 array chips (Illumina) for HN5 and HumanHT-12 v4 array chips for FaDu samples (Illumina).

Data normalization was performed by the AGRF. Briefly, raw signal intensity values were subjected to variance stabilization transformation including background correction, log2 transformation and variance stabilization using the R Bioconductor ‘lumiR’ package (http://www.bioconductor.org). Subsequently, data were quantile normalized in Partek Genomics Suite 6.5 (Partek, Inc).

Parametric two-tailed Student’s *t*-test was used to calculate significance of variation, fold change was calculated as mean ratio. Probes with an unadjusted p-value of 0.05 or less and an absolute fold change of 1.5 or more were defined as differentially expressed. Gene ontology enrichment analysis was performed on the list of differentially expressed probes in Partek® Genomics Suite 6.5.

Ingenuity Pathway Analysis® (Ingenuity System, Inc) was used to confirm the overrepresentation of putative miR-7 target genes among the microarray list of genes downregulated by miR-7. TargetScan (Version 6.0: November 2011) was used for miR-7 target predictions. Microarray expression data has been deposited in the Gene Expression Omnibus under Accession Number GSE40130.

### Microarray Data Analysis

Clustering and volcano plots that showed distributions of miR-7 induced differential gene expression in HN5 and FaDu microarrays were produced from normalised data using the R ‘graphics’ package [62]. A heat map was produced with the R ‘gplots’ package that showed a comparison of significant differential gene expressions across either HN5 or FaDu microarrays. The Venn diagram was produced using the R ‘vennDiagram’ package. A scatter plot and comparison of the correlation of genes commonly downregulated across FaDu and HN5 was produced using the R ‘graphics’ package. Ingenuity Pathway Analysis® was used to determine the PI3K/Akt pathway targets and cellular functions affected by miR-7 as follows: the significant miR-7 downregulated gene lists from HN5 and FaDu were uploaded, analyzed using a core analysis and corresponding figures produced with PathDesigner.

### Statistics

All results are presented as mean ± standard deviation (SD). Statistical significance was calculated using the Student’s *t* test (two-tailed, unpaired) and the level of significance was set at p<0.05. All samples for immunoblotting were loaded in duplicate to validate equal loading of protein. Statistical analysis of RT-qPCR data was performed using GenEx software (MultiD). Normality of data was confirmed using the Kolmogorov-Smirnov test.

Erlotinib sensitivity (EC_50_) was calculated using GraphPad Prism software (GraphPad Software). An erlotinib sensitive cell line was defined as having an EC_50_ below 5 µM, based on published data [63] and results higher than this deemed erlotinib resistant. Synergy between the combination of miR-7 and erlotinib was evaluated using the Bliss additivism model [34], using the formula: E_bliss_ = E_A_+E_B_–E_A_×E_B_. E_A_ was defined as the fractional inhibition obtained by miR-7 alone and E_B_ defined as the fractional inhibition obtained by erlotinib alone. E_bliss_ was the fractional inhibition that would be expected if the combination of miR-7 and erlotinib was additive. If the experimentally measured fractional inhibition was greater than E_bliss_, the combination of miR-7 and erlotinib was said to be synergistic.

## Supporting Information

Figure S1 **miR-7 expression and EGFR/Akt expression and activity across HN5/miR-7 stable clones.** (A) TaqMan RT-qPCR analysis of miR-7 expression in multiple HN5 clones with stable expression of miR-7 (clones 38, 39, 40) or miR-NC (clone 2). Data was normalized to U44 snRNA expression and expressed relative to HN5 miR-NC clone 2. (B) Western blotting analysis of EGFR, Akt and P-Akt levels in HN5 clones with stable expression of miR-7 (clones 38, 39, 40). β-actin is included as a loading control. Error bars represent standard deviations. All data are representative of three independent experiments. *, p<0.01, miR-7 vs miR-NC.
(TIF)

Figure S2 **Transient miR-7 expression inhibits HNC xenograft tumor formation** ***in vivo*** **.** (A) HN5 tumor xenograft formation 10 d after subcutaneous injection of HN5 cells that had been transiently transfected for 24 h with miR-7, miR-NC, or vehicle (LF2000) only into nude mice. Mean tumor volumes (mm^3^) are plotted at 10 d (d). (B) Representative photographs of tumor xenografts for cells with transient miR-NC expression (left) and miR-7 expression (right). Error bars represent standard deviations. *, p<0.01, miR-7 vs miR-NC.
(TIF)

Figure S3 **Synergistic inhibition of cell growth in SCC-25 cells by miR-7 and erlotinib.** Cell titre analysis of SCC-25 cells that were transfected with vehicle only (LF2000), miR-7, or miR-NC for 3 d, and then treated with erlotinib (4 µM) or vehicle (DMSO) for a further 4 d. Data is expressed relative to vehicle-transfected, vehicle-treated SCC-25 cells (LF2000 minus erlotinib, first column).
(TIF)

Figure S4 **RT-qPCR validation of miR-7 microarray targets in HNC cell lines.** HN5 or FaDu cells were transiently transfected with miR-7 or miR-NC for 24 h, total RNA isolated and RT-qPCR analysis performed for RAF1 (A) and TGFA (B) mRNA expression. Data was normalized to GAPDH mRNA expression and expressed relative to miR-NC-transfected cells. Error bars represent standard deviations. All data are representative of three independent experiments. *, p<0.01, miR-7 vs miR-NC; **, p<0.05, miR-7 vs miR-NC.
(TIF)

Figure S5 **Induction of E-cadherin expression in FaDu cells by miR-7.** Western blotting analysis of E-cadherin expression in FaDu cells that were transfected with miR-7 or miR-NC for 3 d. β-actin is included as a loading control.
(TIF)

Table S1 **mRNAs downregulated by miR-7 in HN5 cells.** List of mRNAs identified by microarray analysis as significantly downregulated in HN5 cells 24 h after transient transfection with miR-7 relative to miR-NC.
(DOCX)

Table S2 **mRNAs downregulated by miR-7 in FaDu cells.** List of mRNAs identified by microarray analysis as significantly downregulated in FaDu cells 24 h after transient transfection with miR-7 relative to miR-NC.
(DOCX)

Table S3 **mRNAs downregulated by miR-7 in both HN5 and FaDu cells.** List of mRNAs identified by microarray analysis as significantly downregulated in both HN5 and FaDu cells 24 h after transient transfection with miR-7 relative to miR-NC.
(DOCX)
