# Supplementary figures and images for: Regulation of Epidermal Growth Factor Receptor Signaling and Erlotinib Sensitivity in Head and Neck Cancer Cells by miR-7
Source: PLoS One. 2012 Oct 24;7(10):e47067. doi: 10.1371/journal.pone.0047067 (PMC3480380; doi:10.1371/journal.pone.0047067)

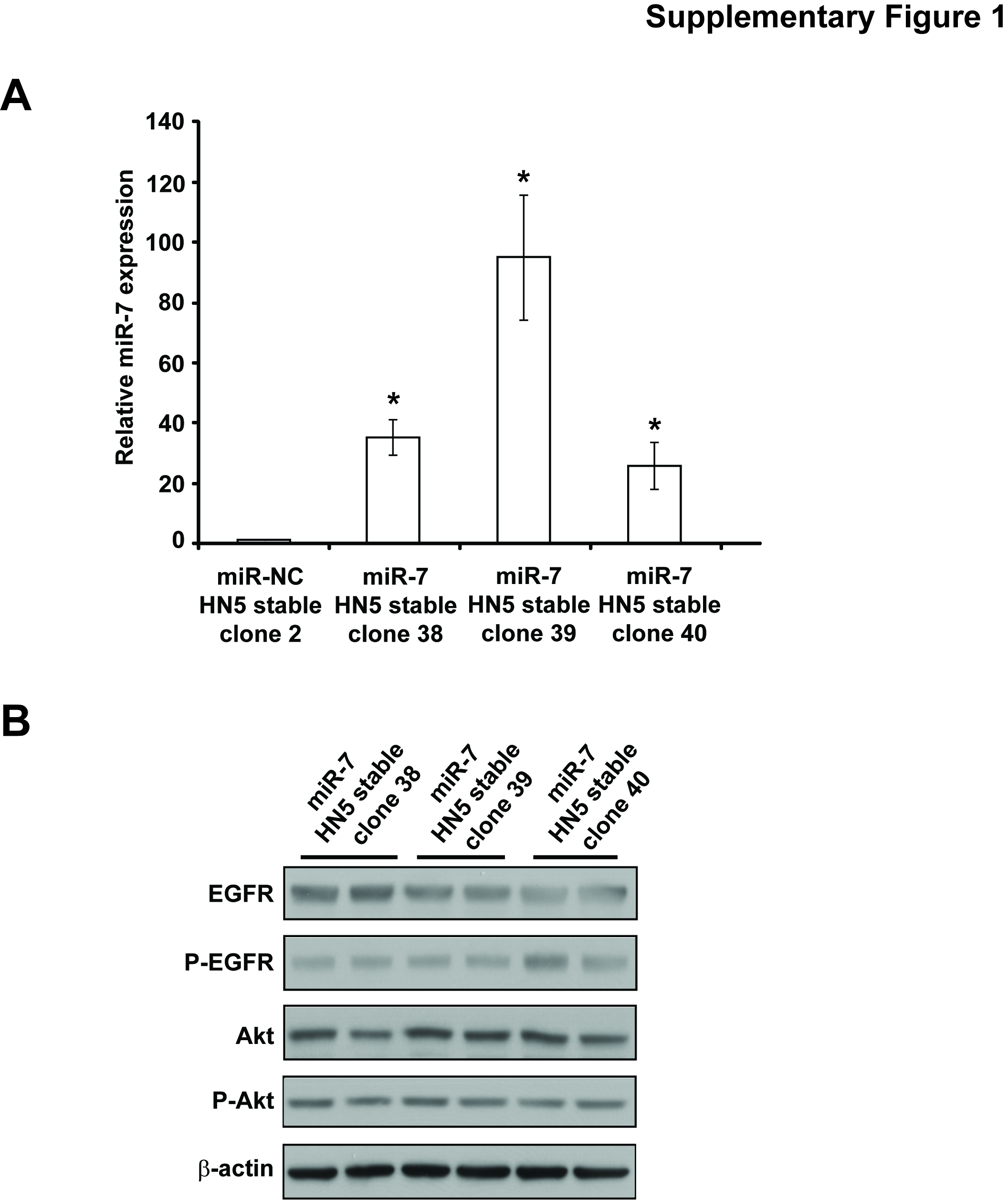

Supplement: Figure S1 — miR-7 expression and EGFR/Akt expression and activity across HN5/miR-7 stable clones. (A) TaqMan RT-qPCR analysis of miR-7 expression in multiple HN5 clones with stable expression of miR-7 (clones 38, 39, 40) or miR-NC (clone 2). Data was normalized to U44 snRNA expression and expressed relative to HN5 miR-NC clone 2. (B) Western blotting analysis of EGFR, Akt and P-Akt levels in HN5 clones with stable expression of miR-7 (clones 38, 39, 40). β-actin is included as a loading control. Error bars represent standard deviations. All data are representative of three independent experiments. *, p<0.01, miR-7 vs miR-NC. (TIF) [file pone.0047067.s001.tif]

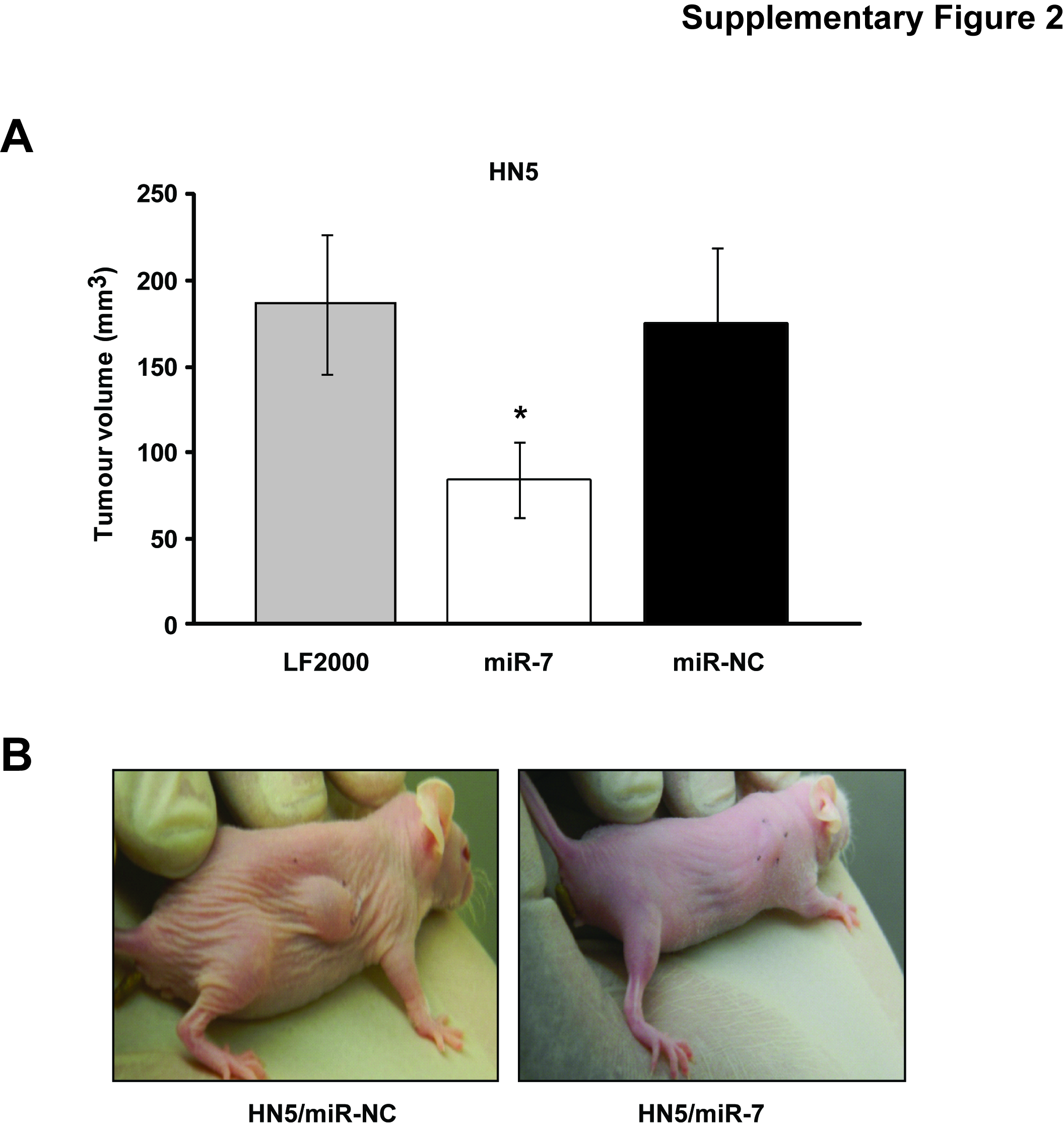

Supplement: Figure S2 — Transient miR-7 expression inhibits HNC xenograft tumor formation in vivo . (A) HN5 tumor xenograft formation 10 d after subcutaneous injection of HN5 cells that had been transiently transfected for 24 h with miR-7, miR-NC, or vehicle (LF2000) only into nude mice. Mean tumor volumes (mm3) are plotted at 10 d (d). (B) Representative photographs of tumor xenografts for cells with transient miR-NC expression (left) and miR-7 expression (right). Error bars represent standard deviations. *, p<0.01, miR-7 vs miR-NC. (TIF) [file pone.0047067.s002.tif]

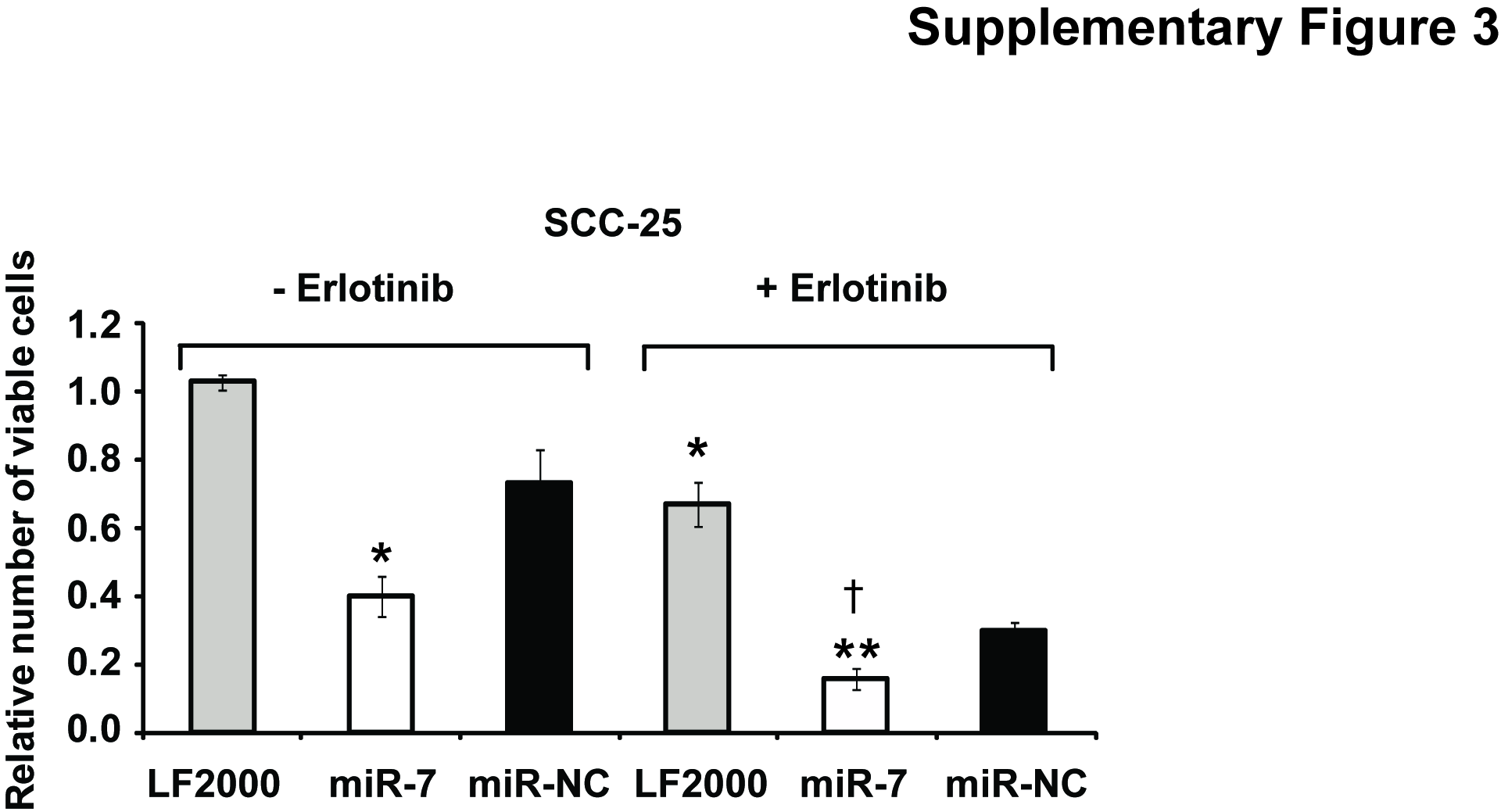

Supplement: Figure S3 — Synergistic inhibition of cell growth in SCC-25 cells by miR-7 and erlotinib. Cell titre analysis of SCC-25 cells that were transfected with vehicle only (LF2000), miR-7, or miR-NC for 3 d, and then treated with erlotinib (4 µM) or vehicle (DMSO) for a further 4 d. Data is expressed relative to vehicle-transfected, vehicle-treated SCC-25 cells (LF2000 minus erlotinib, first column). (TIF) [file pone.0047067.s003.tif]

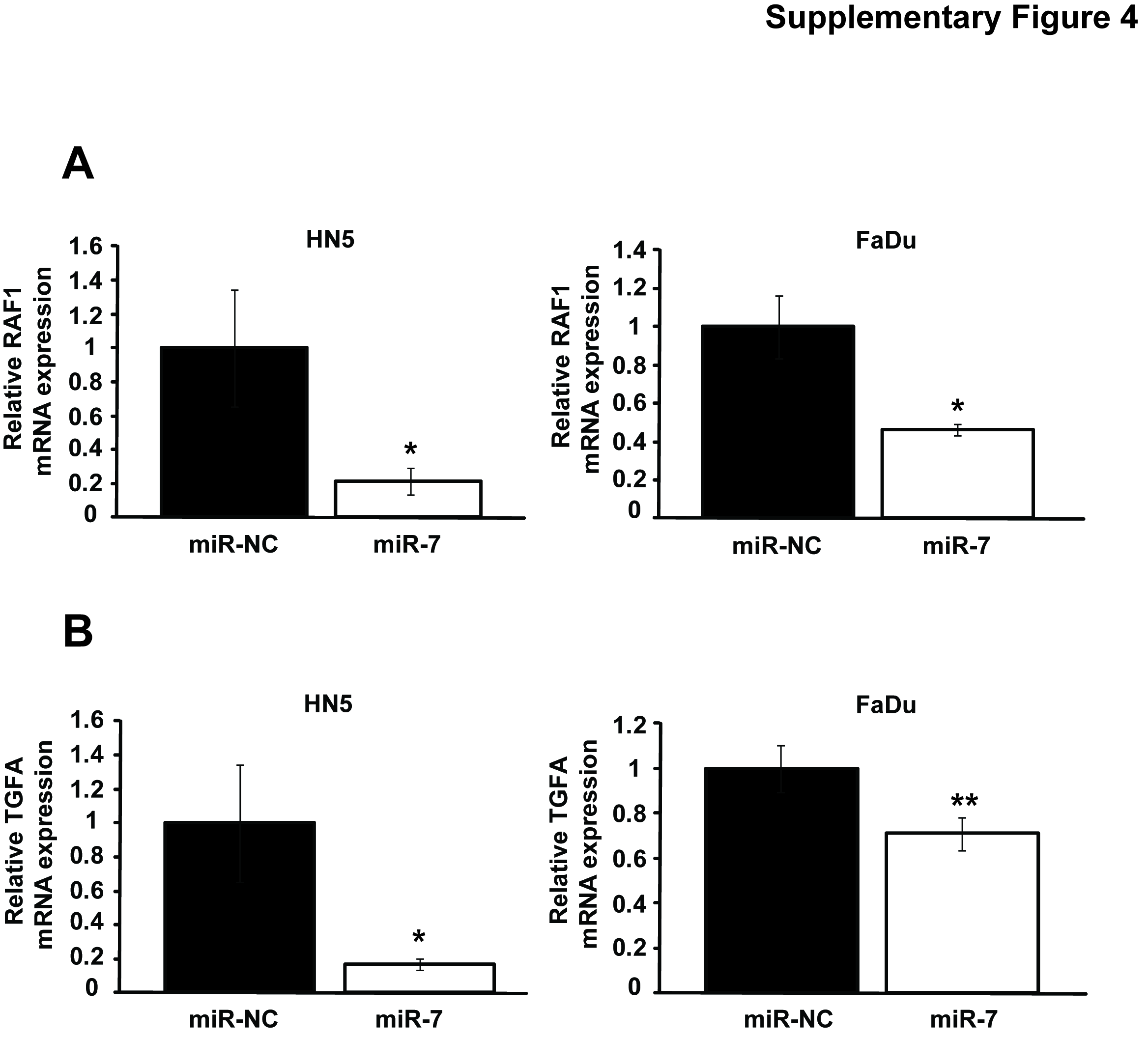

Supplement: Figure S4 — RT-qPCR validation of miR-7 microarray targets in HNC cell lines. HN5 or FaDu cells were transiently transfected with miR-7 or miR-NC for 24 h, total RNA isolated and RT-qPCR analysis performed for RAF1 (A) and TGFA (B) mRNA expression. Data was normalized to GAPDH mRNA expression and expressed relative to miR-NC-transfected cells. Error bars represent standard deviations. All data are representative of three independent experiments. *, p<0.01, miR-7 vs miR-NC; **, p<0.05, miR-7 vs miR-NC. (TIF) [file pone.0047067.s004.tif]

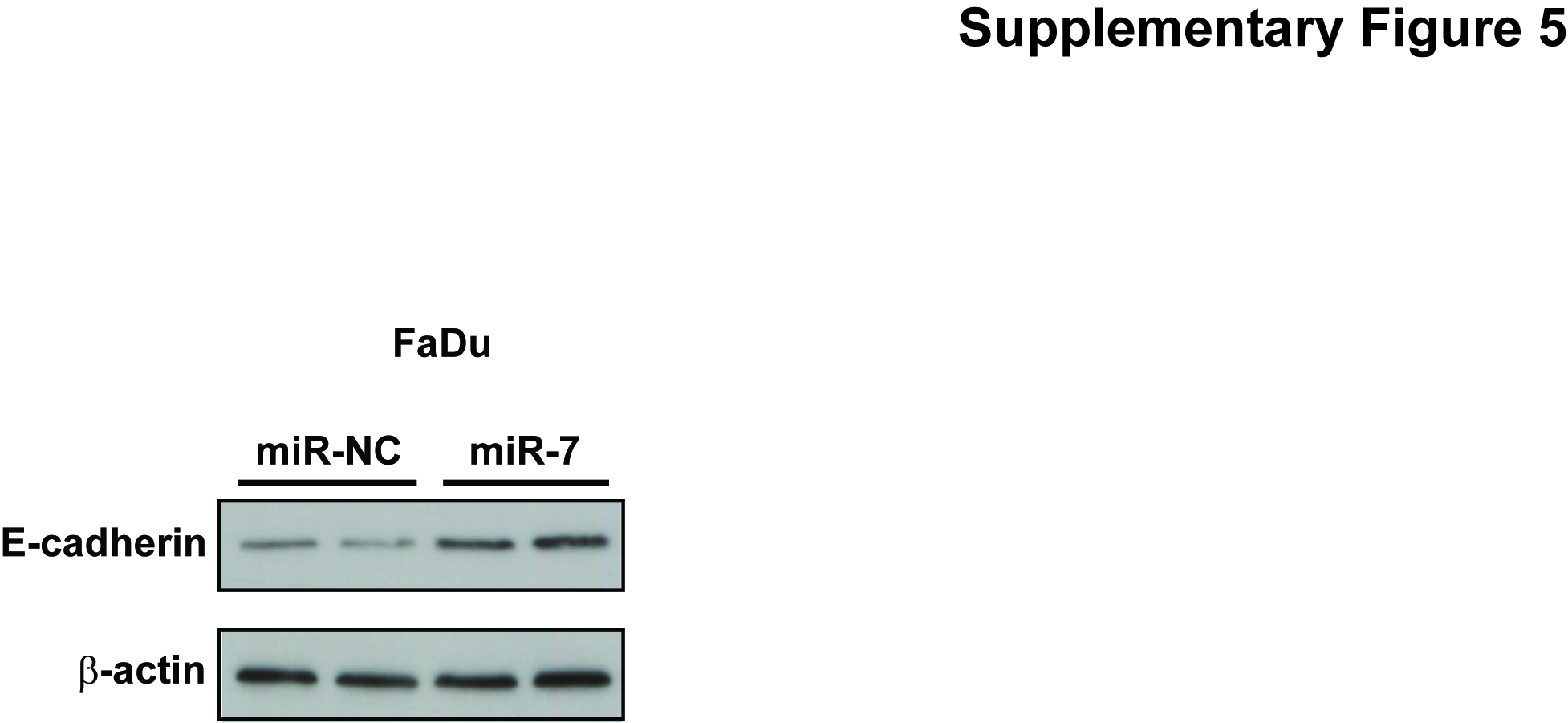

Supplement: Figure S5 — Induction of E-cadherin expression in FaDu cells by miR-7. Western blotting analysis of E-cadherin expression in FaDu cells that were transfected with miR-7 or miR-NC for 3 d. β-actin is included as a loading control. (TIF) [file pone.0047067.s005.tif]
