# Supplementary material for: Regulation of Epidermal Growth Factor Receptor Signaling and Erlotinib Sensitivity in Head and Neck Cancer Cells by miR-7
Source: PLoS One. 2012 Oct 24;7(10):e47067. doi: 10.1371/journal.pone.0047067 (PMC3480380; doi:10.1371/journal.pone.0047067)
Supplement: Table S1 — mRNAs downregulated by miR-7 in HN5 cells. List of mRNAs identified by microarray analysis as significantly downregulated in HN5 cells 24 h after transient transfection with miR-7 relative to miR-NC. (DOCX) [file pone.0047067.s006.docx]

**Supplementary Table 1:** mRNAs downregulated by miR-7 in HN5 cells

| **Gene Symbol** | **p-value** | **Fold-Change** |
| --- | --- | --- |
| AADACL1 | 0.007465 | -1.65391 |
| ACO1 | 0.025078 | -1.70246 |
| ACO2 | 0.007174 | -4.13849 |
| ACSL4 | 0.014456 | -2.22764 |
| ADCY9 | 0.007114 | -1.54369 |
| ADO | 0.018583 | -1.7589 |
| ADPRHL2 | 0.00353 | -1.55476 |
| AGK | 0.004307 | -2.64611 |
| AMMECR1 | 0.031802 | -1.55047 |
| ANKS1A | 0.024151 | -1.67109 |
| ARMC10 | 0.001206 | -1.79324 |
| ATP1B3 | 0.023673 | -1.66208 |
| BCL2L12 | 0.003071 | -3.6053 |
| BIN1 | 0.003361 | -1.91384 |
| BNC1 | 0.011892 | -1.66895 |
| C10orf57 | 0.015041 | -2.20516 |
| C12orf49 | 0.017939 | -1.78836 |
| C14orf151 | 0.037281 | -1.54845 |
| C18orf10 | 0.03026 | -2.32569 |
| C1QTNF6 | 0.013774 | -1.52209 |
| CALM3 | 0.004185 | -2.39904 |
| CALU | 0.009315 | -1.57845 |
| CBL | 0.03114 | -1.52431 |
| CCNE1 | 0.009689 | -1.50773 |
| CGI-96 | 0.027908 | -1.68706 |
| CHEK1 | 0.019397 | -1.67651 |
| CHES1 | 0.001664 | -1.50527 |
| CKAP4 | 0.030776 | -2.78118 |
| CLDN1 | 0.049456 | -1.57057 |
| CMTM4 | 0.009121 | -1.72757 |
| CNN3 | 0.001298 | -2.60267 |
| CNO | 0.0103 | -1.95787 |
| CNTNAP2 | 0.010441 | -1.54699 |
| CPA4 | 0.015613 | -2.3825 |
| CRTAP | 0.010617 | -3.43566 |
| CTDSPL | 0.023965 | -1.90348 |
| DAZAP2 | 0.027065 | -2.01827 |
| DNAJC15 | 0.014636 | -1.69596 |
| DUSP7 | 0.035177 | -1.63458 |
| DYM | 0.010411 | -2.41659 |
| EGFR | 0.001891 | -3.26581 |
| EHD1 | 0.035907 | -3.68397 |
| EIF2AK1 | 0.013058 | -1.59115 |
| EIF2S3 | 0.012332 | -1.93709 |
| ELK1 | 0.004783 | -1.85531 |
| EXOSC2 | 0.016573 | -1.88965 |
| EXTL3 | 0.0195 | -1.73312 |
| FAM173B | 0.007418 | -1.77089 |
| FAM82A2 | 0.015312 | -1.67899 |
| FAM83A | 0.01503 | -1.83503 |
| FAM83D | 0.04981 | -1.69954 |
| FLJ35801 | 0.04182 | -1.50815 |
| GALE | 0.00662 | -1.55723 |
| GLO1 | 0.03192 | -2.09271 |
| GLS | 0.000156 | -2.80636 |
| GLTPD1 | 0.007449 | -1.82827 |
| GNA12 | 0.025218 | -1.54917 |
| HAS3 | 0.049555 | -1.57366 |
| HBEGF | 0.027494 | -1.50462 |
| HIP1 | 0.003082 | -1.70908 |
| IGSF3 | 0.048681 | -1.82798 |
| IKBKE | 0.003861 | -1.55762 |
| IL1B | 0.005045 | -2.51534 |
| IL7R | 0.039631 | -1.56438 |
| IL8 | 0.002907 | -2.0305 |
| ISY1 | 0.049775 | -1.5841 |
| ITFG2 | 0.008267 | -1.50144 |
| KBTBD2 | 0.015834 | -1.62587 |
| KIAA0247 | 0.004615 | -1.90035 |
| KIAA0251 | 0.034683 | -1.77287 |
| KIAA0746 | 0.006096 | -1.89028 |
| KIAA1160 | 0.044461 | -1.57437 |
| LBH | 0.049749 | -1.66036 |
| LEMD3 | 0.006313 | -1.57682 |
| LITAF | 0.001465 | -2.4437 |
| LOC285176 | 0.015237 | -1.54238 |
| LOC401238 | 0.0318 | -1.92678 |
| LOC646675 | 0.004823 | -1.88722 |
| LOC727761 | 0.037971 | -1.96449 |
| LOC727825 | 0.024271 | -1.97386 |
| LYPD3 | 0.007573 | -2.7478 |
| MAML1 | 0.033921 | -1.68395 |
| MAZ | 0.008954 | -1.52528 |
| METRNL | 0.012182 | -1.61217 |
| MFSD5 | 0.033303 | -1.6695 |
| MGC2752 | 0.049796 | -1.5623 |
| MGLL | 0.007898 | -2.42584 |
| MICALL1 | 0.013019 | -1.8185 |
| MSC | 0.044012 | -1.50121 |
| NDE1 | 0.005613 | -1.53042 |
| NDFIP2 | 0.031512 | -1.73524 |
| NFKB1 | 0.042932 | -1.56967 |
| NR1H2 | 0.014028 | -2.51427 |
| NUDCD3 | 0.027997 | -1.98278 |
| OPRS1 | 0.035035 | -1.61431 |
| ORAI1 | 0.01321 | -2.09961 |
| PAK1 | 0.009026 | -1.80668 |
| PHC2 | 0.045561 | -1.58985 |
| PIK3CD | 0.009359 | -2.03196 |
| PKMYT1 | 0.030369 | -1.65634 |
| PKP1 | 0.019587 | -1.73071 |
| PKP2 | 0.034369 | -1.70988 |
| PLEC1 | 0.010043 | -2.75472 |
| PLLP | 0.003331 | -1.90276 |
| PLXNA1 | 0.027938 | -1.89485 |
| POLE4 | 0.008039 | -4.70033 |
| PORCN | 0.040352 | -1.9212 |
| PPRC1 | 0.029754 | -1.79226 |
| PQLC1 | 0.010607 | -2.24041 |
| PRKRIR | 0.022198 | -2.13412 |
| PRMT2 | 0.002785 | -2.23882 |
| PSME3 | 0.000525 | -3.33815 |
| PUS1 | 0.028766 | -1.55667 |
| RAB11FIP5 | 1.74E-05 | -2.22608 |
| RAB5B | 0.012329 | -1.71285 |
| RAF1 | 0.008206 | -2.64895 |
| RELA | 0.034571 | -1.80072 |
| REXO1 | 0.037526 | -1.77201 |
| RHBDF2 | 0.031351 | -1.70794 |
| RNF38 | 0.02402 | -1.63155 |
| RNF40 | 0.049386 | -1.54768 |
| RNF5 | 0.029473 | -2.20098 |
| RNF5P1 | 0.009425 | -2.74543 |
| RRP7A | 0.011428 | -1.96664 |
| RSBN1 | 0.043073 | -1.60731 |
| RYK | 0.033164 | -1.88849 |
| SDC4 | 0.002946 | -1.69883 |
| SEPW1 | 0.037599 | -1.9707 |
| SERP1 | 0.024835 | -1.73933 |
| SETD8 | 0.004598 | -2.20516 |
| SFRS4 | 0.002783 | -2.23211 |
| SH3BP4 | 0.025339 | -1.64375 |
| SH3RF2 | 0.005587 | -1.56807 |
| SKP1A | 0.003036 | -1.94275 |
| SLC25A15 | 0.037107 | -1.86234 |
| SLC35A4 | 0.007491 | -1.7651 |
| SLC35A5 | 0.031421 | -1.59938 |
| SLC39A11 | 0.003627 | -2.72305 |
| SLC39A3 | 0.000498 | -1.65013 |
| SLC45A3 | 0.034457 | -1.52771 |
| SLC6A9 | 0.006426 | -2.27686 |
| SMARCA4 | 0.01665 | -1.514 |
| SMARCD1 | 0.030092 | -2.87353 |
| SNAP29 | 0.004343 | -2.00779 |
| SNCA | 0.021587 | -1.91537 |
| SPATA2 | 0.02446 | -1.64507 |
| SRF | 0.046275 | -1.68996 |
| SRM | 0.048266 | -1.58831 |
| ST3GAL5 | 0.018742 | -1.80845 |
| STX5 | 0.0119 | -1.68974 |
| STX6 | 0.00415 | -1.71929 |
| SYK | 0.006235 | -1.55979 |
| TBC1D2B | 0.034639 | -2.22343 |
| TGFA | 0.024852 | -2.16319 |
| TGOLN2 | 0.008073 | -1.69328 |
| TIPARP | 0.045329 | -1.77723 |
| TMED9 | 0.017241 | -1.72745 |
| TMEM14C | 0.042151 | -1.55818 |
| TMEM184B | 0.032324 | -1.58713 |
| TMEM43 | 0.004681 | -3.29319 |
| TMEM69 | 0.011122 | -1.73625 |
| TMEM97 | 0.04695 | -1.78024 |
| TTLL12 | 0.017948 | -1.75564 |
| TUSC2 | 0.024923 | -1.69878 |
| TYRO3 | 0.019528 | -1.54051 |
| UBE2D4 | 0.002245 | -1.54996 |
| UBE2J1 | 0.005512 | -1.6007 |
| UBE2N | 0.049585 | -1.77136 |
| UBE3C | 0.020141 | -2.13788 |
| UBQLN4 | 0.04997 | -2.12705 |
| UHRF1 | 0.040311 | -2.12065 |
| VGLL4 | 0.027097 | -2.05949 |
| VPS26A | 0.00762 | -2.75664 |
| WDR4 | 0.003311 | -1.69204 |
| WDR72 | 0.001061 | -1.86061 |
| ZDHHC9 | 0.021829 | -2.52794 |
| ZNF395 | 0.017217 | -2.13114 |
| ZNF828 | 0.008483 | -2.37284 |
| ZYX | 0.033837 | -1.79776 |
