# Supplementary material for: Regulation of Epidermal Growth Factor Receptor Signaling and Erlotinib Sensitivity in Head and Neck Cancer Cells by miR-7
Source: PLoS One. 2012 Oct 24;7(10):e47067. doi: 10.1371/journal.pone.0047067 (PMC3480380; doi:10.1371/journal.pone.0047067)
Supplement: Table S2 — mRNAs downregulated by miR-7 in FaDu cells. List of mRNAs identified by microarray analysis as significantly downregulated in FaDu cells 24 h after transient transfection with miR-7 relative to miR-NC. (DOCX) [file pone.0047067.s007.docx]

**Supplementary Table 2:** mRNAs downregulated by miR-7 in FaDu cells

| **Gene Symbol** | **p-value** | **Fold-Change** |
| --- | --- | --- |
| AP3M1 | 3.81E-08 | -2.23018 |
| LOC727761 | 3.05E-07 | -2.3561 |
| HS.538962 | 3.40E-07 | -1.96086 |
| RRP7A | 4.15E-07 | -2.42662 |
| RPL26 | 7.26E-07 | -1.76198 |
| PATL1 | 7.58E-07 | -1.9742 |
| CALM3 | 8.61E-07 | -4.62237 |
| RNF5P1 | 8.96E-07 | -3.80125 |
| AKR7A2 | 9.22E-07 | -2.43591 |
| PSME3 | 9.48E-07 | -5.6208 |
| CNRIP1 | 1.04E-06 | -1.88895 |
| AGK | 1.25E-06 | -2.46034 |
| POLE4 | 1.25E-06 | -8.46889 |
| UBE2D4 | 1.28E-06 | -1.73671 |
| SUPT4H1 | 1.80E-06 | -2.25988 |
| TRIM8 | 1.80E-06 | -2.3766 |
| ZDHHC9 | 2.06E-06 | -4.66844 |
| NBL1 | 2.09E-06 | -1.61532 |
| SMARCD1 | 2.16E-06 | -3.76508 |
| TSEN34 | 2.42E-06 | -2.08609 |
| C1QTNF6 | 2.45E-06 | -2.11529 |
| RNF181 | 2.56E-06 | -3.05807 |
| LOC100134537 | 2.61E-06 | -2.68096 |
| PIGH | 2.70E-06 | -1.85154 |
| TGOLN2 | 2.84E-06 | -2.40751 |
| CRTAP | 2.88E-06 | -3.19729 |
| OLR1 | 2.99E-06 | -1.8781 |
| VPS26A | 3.29E-06 | -2.96325 |
| RUSC1 | 3.61E-06 | -3.71431 |
| GAD1 | 3.64E-06 | -1.94537 |
| YWHAB | 3.85E-06 | -1.85806 |
| CKAP4 | 3.92E-06 | -2.05783 |
| RYK | 4.17E-06 | -1.57731 |
| SKP1 | 4.22E-06 | -1.9557 |
| NR1H2 | 5.32E-06 | -1.99365 |
| C18ORF10 | 5.64E-06 | -2.78414 |
| LOC100133888 | 5.68E-06 | -1.56416 |
| METRNL | 6.23E-06 | -1.55207 |
| SLC39A3 | 6.50E-06 | -2.79065 |
| RPL22 | 7.14E-06 | -2.73081 |
| VAMP8 | 7.14E-06 | -2.91785 |
| WBP2 | 7.37E-06 | -1.74364 |
| CHES1 | 7.48E-06 | -2.14935 |
| PDXDC1 | 7.79E-06 | -2.74444 |
| TMEM134 | 7.84E-06 | -1.82704 |
| GLO1 | 7.96E-06 | -3.58238 |
| LOC100130919 | 7.97E-06 | -3.06637 |
| LEPREL1 | 8.03E-06 | -1.83594 |
| MFSD10 | 8.55E-06 | -2.13367 |
| LITAF | 9.02E-06 | -4.231 |
| LOC389342 | 9.07E-06 | -1.98588 |
| LEMD3 | 9.52E-06 | -1.73486 |
| HS.10862 | 9.88E-06 | -2.98033 |
| IL1B | 1.04E-05 | -2.86183 |
| UBAP1 | 1.08E-05 | -1.82973 |
| ILF3 | 1.10E-05 | -1.80227 |
| STX6 | 1.12E-05 | -1.70733 |
| PIK3R2 | 1.14E-05 | -2.17372 |
| EGFR | 1.15E-05 | -2.13369 |
| ZNF828 | 1.17E-05 | -2.19915 |
| PQLC1 | 1.27E-05 | -1.83389 |
| SLC35A5 | 1.27E-05 | -1.59238 |
| LOC653506 | 1.28E-05 | -2.09718 |
| COG2 | 1.35E-05 | -1.736 |
| PRKRIR | 1.37E-05 | -2.09461 |
| DAZAP2 | 1.40E-05 | -2.28448 |
| TMEM69 | 1.43E-05 | -2.1985 |
| EED | 1.45E-05 | -1.54073 |
| EIF2AK1 | 1.51E-05 | -2.86023 |
| IDE | 1.56E-05 | -1.60038 |
| LOC644761 | 1.56E-05 | -1.74224 |
| SLC35A4 | 1.58E-05 | -2.21237 |
| LOC100131785 | 1.64E-05 | -2.50226 |
| CTDSP2 | 1.65E-05 | -2.12234 |
| RNF114 | 1.67E-05 | -2.34113 |
| SCARB2 | 1.69E-05 | -2.37494 |
| C11ORF24 | 1.74E-05 | -1.59383 |
| NDFIP2 | 1.76E-05 | -1.90022 |
| TMUB2 | 1.76E-05 | -1.55786 |
| BCL2L12 | 1.81E-05 | -3.43613 |
| ATP1B3 | 1.82E-05 | -2.16174 |
| ATG7 | 1.83E-05 | -1.79793 |
| KIAA0251 | 1.93E-05 | -1.52373 |
| XPR1 | 1.96E-05 | -2.32452 |
| MXRA5 | 1.99E-05 | -1.89396 |
| SLC25A39 | 2.04E-05 | -2.6031 |
| NUDCD3 | 2.06E-05 | -1.68525 |
| HMGN4 | 2.13E-05 | -2.29965 |
| UBQLN4 | 2.15E-05 | -3.01569 |
| LOC647597 | 2.18E-05 | -2.50609 |
| ELK1 | 2.21E-05 | -1.99605 |
| RHOD | 2.29E-05 | -1.59405 |
| SLC39A11 | 2.30E-05 | -1.83667 |
| TMEM14D | 2.31E-05 | -2.86703 |
| CDC37 | 2.32E-05 | -1.91485 |
| TMEM43 | 2.33E-05 | -2.52398 |
| PKP2 | 2.35E-05 | -1.54467 |
| NDUFA4 | 2.38E-05 | -3.19253 |
| CNN3 | 2.42E-05 | -2.88184 |
| DYM | 2.44E-05 | -1.7038 |
| ARMC10 | 2.46E-05 | -2.36975 |
| DHCR24 | 2.47E-05 | -2.47699 |
| SERP1 | 2.47E-05 | -1.8776 |
| TMED9 | 2.49E-05 | -2.49419 |
| COMMD7 | 2.64E-05 | -1.88326 |
| EIF2B3 | 2.72E-05 | -1.70935 |
| ACO2 | 2.76E-05 | -3.14949 |
| RCC2 | 2.83E-05 | -3.08535 |
| TOMM6 | 2.85E-05 | -1.50347 |
| HS.475334 | 2.88E-05 | -1.70099 |
| FGFR3 | 2.94E-05 | -1.79115 |
| PACS1 | 3.01E-05 | -1.73155 |
| RFNG | 3.04E-05 | -1.77629 |
| STX5 | 3.05E-05 | -2.20317 |
| LOC100129650 | 3.17E-05 | -1.61023 |
| FAM82A2 | 3.19E-05 | -1.93499 |
| KDM5B | 3.35E-05 | -1.55036 |
| CTDSPL | 3.43E-05 | -1.963 |
| TMEM97 | 3.52E-05 | -1.99165 |
| AADACL1 | 3.53E-05 | -1.9568 |
| EHD1 | 3.82E-05 | -2.35126 |
| RAB11FIP5 | 3.87E-05 | -1.59966 |
| TMEM14C | 3.87E-05 | -1.90955 |
| CHEK1 | 3.92E-05 | -1.85031 |
| PPRC1 | 3.93E-05 | -1.59473 |
| CCND2 | 4.13E-05 | -2.2583 |
| ELF3 | 4.33E-05 | -1.83481 |
| PRMT2 | 4.56E-05 | -2.49336 |
| FAM65A | 4.62E-05 | -1.67209 |
| S100A2 | 4.69E-05 | -1.68036 |
| RSBN1 | 4.85E-05 | -1.6455 |
| CYBASC3 | 4.97E-05 | -1.82916 |
| SNORA33 | 5.05E-05 | -1.87658 |
| HS.538259 | 5.07E-05 | -2.11236 |
| EXOSC2 | 5.12E-05 | -1.58253 |
| SNHG8 | 5.14E-05 | -1.85207 |
| ATF5 | 5.20E-05 | -2.06228 |
| PEX19 | 5.22E-05 | -1.54082 |
| NIF3L1 | 5.30E-05 | -1.54653 |
| ANKS1A | 5.43E-05 | -1.57146 |
| UBE3C | 5.48E-05 | -2.54484 |
| WDR72 | 5.54E-05 | -2.65303 |
| PIK3CB | 5.58E-05 | -1.56044 |
| SIGMAR1 | 5.65E-05 | -1.92008 |
| KLK6 | 5.70E-05 | -1.53191 |
| NUB1 | 5.76E-05 | -1.60786 |
| EIF2S3 | 5.83E-05 | -3.23336 |
| ARHGAP1 | 6.04E-05 | -1.76938 |
| SFRS4 | 6.15E-05 | -1.62469 |
| NQO1 | 6.27E-05 | -1.58085 |
| LYPLA1 | 6.38E-05 | -1.58133 |
| ALG3 | 6.40E-05 | -1.93452 |
| AUP1 | 6.40E-05 | -1.9041 |
| CPA4 | 6.40E-05 | -1.86497 |
| ALDOC | 6.45E-05 | -2.0022 |
| RPL15 | 6.58E-05 | -2.01149 |
| CCNE1 | 6.74E-05 | -1.50923 |
| PAQR4 | 7.17E-05 | -1.89528 |
| TMEM93 | 7.22E-05 | -1.88203 |
| SERPINB6 | 7.30E-05 | -1.62379 |
| PFN2 | 7.50E-05 | -1.67566 |
| ITFG2 | 7.54E-05 | -1.54923 |
| GRINA | 7.56E-05 | -1.5385 |
| HOMER2 | 7.59E-05 | -1.50531 |
| DHCR7 | 7.71E-05 | -1.73998 |
| IL1RN | 7.77E-05 | -1.77493 |
| RPL37A | 7.77E-05 | -1.64008 |
| SCNN1A | 8.44E-05 | -1.7307 |
| LOC100132717 | 8.51E-05 | -1.64713 |
| C10ORF57 | 8.65E-05 | -2.01113 |
| SNAP29 | 8.96E-05 | -1.64904 |
| SKP1A | 9.23E-05 | -2.96965 |
| SLC9A1 | 9.63E-05 | -1.96863 |
| ABLIM1 | 9.68E-05 | -1.53255 |
| SPNS1 | 9.76E-05 | -2.20766 |
| MARCKSL1 | 0.0001 | -1.81834 |
| TXLNA | 0.000101 | -1.68599 |
| SEL1L3 | 0.000103 | -1.55908 |
| TIPARP | 0.000104 | -1.55059 |
| PHF16 | 0.000105 | -1.81759 |
| TCF12 | 0.000105 | -1.61495 |
| POLE3 | 0.000106 | -3.30901 |
| RAF1 | 0.000106 | -2.34177 |
| C20ORF24 | 0.000108 | -1.58027 |
| DNAJC15 | 0.00011 | -1.94803 |
| SLC25A15 | 0.00011 | -2.13888 |
| RNF5 | 0.000111 | -1.84327 |
| KRT6A | 0.000114 | -1.9939 |
| SCAP | 0.000115 | -1.63637 |
| SERPINE2 | 0.000116 | -1.70223 |
| LOC646966 | 0.000117 | -1.5537 |
| MFSD5 | 0.000119 | -1.86326 |
| TMEM184B | 0.00012 | -1.99084 |
| ALDH1A3 | 0.00012 | -2.0795 |
| WDR36 | 0.00012 | -1.56112 |
| AIF1L | 0.000122 | -1.85714 |
| RPL34 | 0.000122 | -1.52577 |
| ARF4 | 0.000124 | -1.73159 |
| SRF | 0.000127 | -1.76712 |
| CMTM4 | 0.000128 | -1.72268 |
| GSTT1 | 0.000128 | -1.52025 |
| PIP4K2C | 0.00013 | -1.80603 |
| SNORA61 | 0.00013 | -1.93473 |
| NOL6 | 0.000131 | -1.69145 |
| UBE2N | 0.000132 | -1.62716 |
| IGFL1 | 0.000134 | -1.75222 |
| CRTC3 | 0.000135 | -1.91856 |
| CAV1 | 0.000137 | -1.86956 |
| LOC728059 | 0.000138 | -3.38139 |
| PTGES | 0.000139 | -1.51317 |
| LANCL1 | 0.00014 | -2.23474 |
| UHRF1 | 0.000141 | -1.92639 |
| SNORA57 | 0.000146 | -2.01425 |
| MPDU1 | 0.000148 | -1.6323 |
| UBXN2B | 0.000152 | -1.54773 |
| C16ORF35 | 0.000158 | -1.76863 |
| ID1 | 0.000158 | -1.53652 |
| CNO | 0.000164 | -1.70269 |
| TGFA | 0.000165 | -1.63213 |
| TMEM14B | 0.000166 | -2.7178 |
| ATP2C1 | 0.000166 | -1.54283 |
| VGLL4 | 0.00017 | -1.86276 |
| CSNK2A2 | 0.00017 | -1.57113 |
| SNORD104 | 0.00017 | -2.02607 |
| UTP14A | 0.000175 | -1.55968 |
| KIAA0194 | 0.000176 | -1.50447 |
| LOC644363 | 0.000178 | -2.03745 |
| C1ORF116 | 0.000178 | -1.76978 |
| UBE2J1 | 0.00018 | -1.6639 |
| HIATL1 | 0.000182 | -1.83575 |
| C9ORF86 | 0.000187 | -1.51551 |
| PIGS | 0.000199 | -1.5043 |
| LOC390345 | 0.0002 | -1.63388 |
| C11ORF48 | 0.000201 | -1.51479 |
| LOC389137 | 0.000202 | -1.99338 |
| CNOT8 | 0.000209 | -2.14585 |
| MT1E | 0.00021 | -1.51012 |
| HCP5 | 0.000216 | -1.55316 |
| NCLN | 0.000221 | -1.52446 |
| DUSP23 | 0.000221 | -1.73844 |
| PRICKLE4 | 0.000222 | -1.5938 |
| LOC100127982 | 0.000222 | -1.93728 |
| SNHG4 | 0.000226 | -1.5422 |
| SETD8 | 0.000226 | -2.19534 |
| SNORA24 | 0.000228 | -1.73075 |
| FAM89B | 0.000232 | -1.60354 |
| TMEM150A | 0.000235 | -1.58134 |
| WDR40A | 0.000235 | -1.63432 |
| ACSL4 | 0.000236 | -1.67315 |
| MIF4GD | 0.000236 | -1.73265 |
| RHBDF2 | 0.000242 | -1.50272 |
| IPO11 | 0.000243 | -1.53478 |
| RAB5B | 0.000245 | -1.97863 |
| SSU72 | 0.000261 | -1.62349 |
| MMP13 | 0.000269 | -1.67861 |
| OXSR1 | 0.00027 | -1.83364 |
| KRT80 | 0.000273 | -1.60667 |
| EEF1A1 | 0.000274 | -1.62877 |
| IMPDH2 | 0.000278 | -1.64981 |
| USP39 | 0.000302 | -1.5001 |
| LOC100130511 | 0.000304 | -1.90499 |
| PKMYT1 | 0.000306 | -1.60713 |
| VPS26 | 0.000312 | -1.67256 |
| SH3BP4 | 0.000328 | -1.86901 |
| SLC29A2 | 0.00033 | -1.65002 |
| C20ORF4 | 0.00035 | -1.65142 |
| TBCD | 0.000351 | -1.59159 |
| SHISA5 | 0.000357 | -2.17189 |
| FST | 0.00038 | -1.59035 |
| TNFAIP2 | 0.000387 | -1.75603 |
| LOC649970 | 0.00039 | -1.58845 |
| ADPRHL2 | 0.00039 | -2.02663 |
| APLP2 | 0.000404 | -2.11416 |
| CAPZA1 | 0.000422 | -2.30077 |
| ZDHHC16 | 0.000452 | -1.89619 |
| NFIA | 0.000477 | -1.71253 |
| SNHG1 | 0.000486 | -1.50765 |
| HS.127310 | 0.00049 | -1.50309 |
| SEPT2 | 0.000497 | -1.58843 |
| SDHC | 0.000498 | -1.55051 |
| ZNF395 | 0.000505 | -1.53281 |
| ANKFY1 | 0.000514 | -1.54197 |
| LOC400948 | 0.00052 | -1.57331 |
| MRPL10 | 0.000521 | -1.59721 |
| PES1 | 0.000541 | -1.51783 |
| C6ORF66 | 0.000547 | -1.51888 |
| LOC646817 | 0.000557 | -1.54576 |
| ZYX | 0.000562 | -1.78902 |
| BCL3 | 0.000571 | -1.64912 |
| LOC646753 | 0.000579 | -1.52117 |
| AKR7A3 | 0.00062 | -1.62623 |
| CGI-96 | 0.000627 | -1.61841 |
| FAM83A | 0.000633 | -1.53898 |
| HS.430851 | 0.000635 | -1.608 |
| HECTD3 | 0.000641 | -1.55716 |
| H19 | 0.000643 | -1.77941 |
| ADO | 0.000653 | -1.70899 |
| SDC4 | 0.000653 | -1.68661 |
| TSGA14 | 0.000659 | -1.71981 |
| RPS19BP1 | 0.000679 | -2.06937 |
| DHX33 | 0.00069 | -1.5055 |
| LOC100128731 | 0.000696 | -1.56237 |
| TTLL12 | 0.000703 | -1.85327 |
| SERF2 | 0.000714 | -1.92328 |
| PAK2 | 0.000714 | -1.56657 |
| LOC100130707 | 0.000738 | -1.54246 |
| SRM | 0.000746 | -1.707 |
| LPCAT1 | 0.000766 | -1.79392 |
| LOC374395 | 0.000789 | -1.63147 |
| NSMAF | 0.000807 | -1.61034 |
| DSCR3 | 0.000809 | -1.69786 |
| ATP9A | 0.000837 | -1.51989 |
| CDK7 | 0.000839 | -1.57888 |
| LOC651397 | 0.000889 | -1.52808 |
| PTK2 | 0.000903 | -1.5863 |
| LOC391126 | 0.000913 | -1.7552 |
| TRIB1 | 0.000919 | -1.54593 |
| NXT2 | 0.000989 | -1.52643 |
| PCSK7 | 0.00109 | -1.52898 |
| PCNXL3 | 0.00113 | -1.69926 |
| LOC441073 | 0.001205 | -1.61687 |
| TUFT1 | 0.001282 | -1.50303 |
| ARHGAP23 | 0.001282 | -1.57174 |
| SNORA27 | 0.001288 | -1.72648 |
| OGT | 0.001327 | -1.51923 |
| RSPRY1 | 0.001359 | -1.53262 |
| UBN1 | 0.00138 | -1.551 |
| TMEM219 | 0.001431 | -1.59346 |
| DCAF7 | 0.001489 | -1.74419 |
| PURB | 0.001583 | -1.59671 |
| SLC35B2 | 0.001668 | -1.60375 |
| SLC25A10 | 0.001676 | -1.58302 |
| LOC647000 | 0.001757 | -1.56428 |
| LOC100132547 | 0.001791 | -1.72938 |
| TAP2 | 0.001995 | -1.68041 |
| MGAT2 | 0.002075 | -1.58757 |
| LGALS3BP | 0.00216 | -1.61349 |
| STMN3 | 0.002242 | -1.5064 |
| GALE | 0.002317 | -1.54195 |
| KPNA6 | 0.002335 | -1.63384 |
| RAC1 | 0.002382 | -1.50745 |
| SNORD80 | 0.002451 | -1.79813 |
| KRT18P13 | 0.002486 | -1.81567 |
| PPARBP | 0.002758 | -1.54681 |
| TMED10 | 0.002786 | -1.68448 |
| LAMC2 | 0.00343 | -1.63634 |
| CALU | 0.003466 | -1.51879 |
| VDAC1 | 0.003496 | -1.60295 |
| JAG2 | 0.003537 | -1.53459 |
| VPS25 | 0.004189 | -1.53426 |
| LOC399965 | 0.004224 | -1.5714 |
| SRRM2 | 0.004772 | -1.57389 |
| TAPBP | 0.006058 | -1.60582 |
| KRT13 | 0.006545 | -1.56226 |
| CCDC76 | 0.0076 | -1.53668 |
| NEDD8 | 0.008369 | -1.65185 |
| FRMD8 | 0.008893 | -1.59276 |
