# Supplementary material for: Regulation of Epidermal Growth Factor Receptor Signaling and Erlotinib Sensitivity in Head and Neck Cancer Cells by miR-7
Source: PLoS One. 2012 Oct 24;7(10):e47067. doi: 10.1371/journal.pone.0047067 (PMC3480380; doi:10.1371/journal.pone.0047067)
Supplement: Table S3 — mRNAs downregulated by miR-7 in both HN5 and FaDu cells. List of mRNAs identified by microarray analysis as significantly downregulated in both HN5 and FaDu cells 24 h after transient transfection with miR-7 relative to miR-NC. (DOCX) [file pone.0047067.s008.docx]

**Supplementary Table 3:** mRNAs commonly downregulated by miR-7 in both HN5 and FaDu cells

|  | **FaDu** | | **HN5** | |
| --- | --- | --- | --- | --- |
| **Gene Symbol** | **p-value** | **Fold-Change** | **p-value** | **Fold-Change** |
| AADACL1 | 3.53E-05 | -1.9568 | 0.007465 | -1.65391 |
| ACO2 | 2.76E-05 | -3.14949 | 0.007174 | -4.13849 |
| ACSL4 | 0.000236 | -1.67315 | 0.014456 | -2.22764 |
| ADO | 0.000653 | -1.70899 | 0.018583 | -1.7589 |
| ADPRHL2 | 0.00039 | -2.02663 | 0.00353 | -1.55476 |
| AGK | 1.25E-06 | -2.46034 | 0.004307 | -2.64611 |
| ANKS1A | 5.43E-05 | -1.57146 | 0.024151 | -1.67109 |
| ARMC10 | 2.46E-05 | -2.36975 | 0.001206 | -1.79324 |
| BCL2L12 | 1.81E-05 | -3.43613 | 0.003071 | -3.6053 |
| C1QTNF6 | 2.45E-06 | -2.11529 | 0.013774 | -1.52209 |
| CALM3 | 8.61E-07 | -4.62237 | 0.004185 | -2.39904 |
| CALU | 0.003466 | -1.51879 | 0.009315 | -1.57845 |
| CCNE1 | 6.74E-05 | -1.50923 | 0.009689 | -1.50773 |
| CGI-96 | 0.000627 | -1.61841 | 0.027908 | -1.68706 |
| CHEK1 | 3.92E-05 | -1.85031 | 0.019397 | -1.67651 |
| CHES1 | 7.48E-06 | -2.14935 | 0.001664 | -1.50527 |
| CKAP4 | 3.92E-06 | -2.05783 | 0.030776 | -2.78118 |
| CMTM4 | 0.000128 | -1.72268 | 0.009121 | -1.72757 |
| CNN3 | 2.42E-05 | -2.88184 | 0.001298 | -2.60267 |
| CNO | 0.000164 | -1.70269 | 0.0103 | -1.95787 |
| CPA4 | 6.40E-05 | -1.86497 | 0.015613 | -2.3825 |
| CRTAP | 2.88E-06 | -3.19729 | 0.010617 | -3.43566 |
| CTDSPL | 3.43E-05 | -1.963 | 0.023965 | -1.90348 |
| DAZAP2 | 1.40E-05 | -2.28448 | 0.027065 | -2.01827 |
| DNAJC15 | 0.00011 | -1.94803 | 0.014636 | -1.69596 |
| DYM | 2.44E-05 | -1.7038 | 0.010411 | -2.41659 |
| EGFR | 1.15E-05 | -2.13369 | 0.001891 | -3.26581 |
| EHD1 | 3.82E-05 | -2.35126 | 0.035907 | -3.68397 |
| EIF2AK1 | 1.51E-05 | -2.86023 | 0.013058 | -1.59115 |
| EIF2S3 | 5.83E-05 | -3.23336 | 0.012332 | -1.93709 |
| ELK1 | 2.21E-05 | -1.99605 | 0.004783 | -1.85531 |
| EXOSC2 | 5.12E-05 | -1.58253 | 0.016573 | -1.88965 |
| FAM82A2 | 3.19E-05 | -1.93499 | 0.015312 | -1.67899 |
| FAM83A | 0.000633 | -1.53898 | 0.01503 | -1.83503 |
| GALE | 0.002317 | -1.54195 | 0.00662 | -1.55723 |
| GLO1 | 7.96E-06 | -3.58238 | 0.03192 | -2.09271 |
| IL1B | 1.04E-05 | -2.86183 | 0.005045 | -2.51534 |
| ITFG2 | 7.54E-05 | -1.54923 | 0.008267 | -1.50144 |
| KIAA0251 | 1.93E-05 | -1.52373 | 0.034683 | -1.77287 |
| LEMD3 | 9.52E-06 | -1.73486 | 0.006313 | -1.57682 |
| LITAF | 9.02E-06 | -4.231 | 0.001465 | -2.4437 |
| LOC727761 | 3.05E-07 | -2.3561 | 0.037971 | -1.96449 |
| METRNL | 6.23E-06 | -1.55207 | 0.012182 | -1.61217 |
| MFSD5 | 0.000119 | -1.86326 | 0.033303 | -1.6695 |
| NDFIP2 | 1.76E-05 | -1.90022 | 0.031512 | -1.73524 |
| NR1H2 | 5.32E-06 | -1.99365 | 0.014028 | -2.51427 |
| NUDCD3 | 2.06E-05 | -1.68525 | 0.027997 | -1.98278 |
| PKMYT1 | 0.000306 | -1.60713 | 0.030369 | -1.65634 |
| PKP2 | 2.35E-05 | -1.54467 | 0.034369 | -1.70988 |
| POLE4 | 1.25E-06 | -8.46889 | 0.008039 | -4.70033 |
| PPRC1 | 3.93E-05 | -1.59473 | 0.029754 | -1.79226 |
| PQLC1 | 1.27E-05 | -1.83389 | 0.010607 | -2.24041 |
| PRKRIR | 1.37E-05 | -2.09461 | 0.022198 | -2.13412 |
| PRMT2 | 4.56E-05 | -2.49336 | 0.002785 | -2.23882 |
| PSME3 | 9.48E-07 | -5.6208 | 0.000525 | -3.33815 |
| RAB11FIP5 | 3.87E-05 | -1.59966 | 1.74E-05 | -2.22608 |
| RAB5B | 0.000245 | -1.97863 | 0.012329 | -1.71285 |
| RAF1 | 0.000106 | -2.34177 | 0.008206 | -2.64895 |
| RHBDF2 | 0.000242 | -1.50272 | 0.031351 | -1.70794 |
| RNF5 | 0.000111 | -1.84327 | 0.029473 | -2.20098 |
| RNF5P1 | 8.96E-07 | -3.80125 | 0.009425 | -2.74543 |
| RRP7A | 4.15E-07 | -2.42662 | 0.011428 | -1.96664 |
| RSBN1 | 4.85E-05 | -1.6455 | 0.043073 | -1.60731 |
| SDC4 | 0.000653 | -1.68661 | 0.002946 | -1.69883 |
| SERP1 | 2.47E-05 | -1.8776 | 0.024835 | -1.73933 |
| SETD8 | 0.000226 | -2.19534 | 0.004598 | -2.20516 |
| SFRS4 | 6.15E-05 | -1.62469 | 0.002783 | -2.23211 |
| SH3BP4 | 0.000328 | -1.86901 | 0.025339 | -1.64375 |
| SKP1A | 9.23E-05 | -2.96965 | 0.003036 | -1.94275 |
| SLC25A15 | 0.00011 | -2.13888 | 0.037107 | -1.86234 |
| SLC35A4 | 1.58E-05 | -2.21237 | 0.007491 | -1.7651 |
| SLC35A5 | 1.27E-05 | -1.59238 | 0.031421 | -1.59938 |
| SLC39A11 | 2.30E-05 | -1.83667 | 0.003627 | -2.72305 |
| SLC39A3 | 6.50E-06 | -2.79065 | 0.000498 | -1.65013 |
| SMARCD1 | 2.16E-06 | -3.76508 | 0.030092 | -2.87353 |
| SNAP29 | 8.96E-05 | -1.64904 | 0.004343 | -2.00779 |
| SRF | 0.000127 | -1.76712 | 0.046275 | -1.68996 |
| SRM | 0.000746 | -1.707 | 0.048266 | -1.58831 |
| STX5 | 3.05E-05 | -2.20317 | 0.0119 | -1.68974 |
| STX6 | 1.12E-05 | -1.70733 | 0.00415 | -1.71929 |
| TGFA | 0.000165 | -1.63213 | 0.024852 | -2.16319 |
| TGOLN2 | 2.84E-06 | -2.40751 | 0.008073 | -1.69328 |
| TIPARP | 0.000104 | -1.55059 | 0.045329 | -1.77723 |
| TMED9 | 2.49E-05 | -2.49419 | 0.017241 | -1.72745 |
| TMEM14C | 3.87E-05 | -1.90955 | 0.042151 | -1.55818 |
| TMEM184B | 0.00012 | -1.99084 | 0.032324 | -1.58713 |
| TMEM43 | 2.33E-05 | -2.52398 | 0.004681 | -3.29319 |
| TMEM69 | 1.43E-05 | -2.1985 | 0.011122 | -1.73625 |
| TMEM97 | 3.52E-05 | -1.99165 | 0.04695 | -1.78024 |
| TTLL12 | 0.000703 | -1.85327 | 0.017948 | -1.75564 |
| UBE2D4 | 1.28E-06 | -1.73671 | 0.002245 | -1.54996 |
| UBE2J1 | 0.00018 | -1.6639 | 0.005512 | -1.6007 |
| UBE2N | 0.000132 | -1.62716 | 0.049585 | -1.77136 |
| UBE3C | 5.48E-05 | -2.54484 | 0.020141 | -2.13788 |
| UBQLN4 | 2.15E-05 | -3.01569 | 0.04997 | -2.12705 |
| UHRF1 | 0.000141 | -1.92639 | 0.040311 | -2.12065 |
| VGLL4 | 0.00017 | -1.86276 | 0.027097 | -2.05949 |
| VPS26A | 3.29E-06 | -2.96325 | 0.00762 | -2.75664 |
| WDR72 | 5.54E-05 | -2.65303 | 0.001061 | -1.86061 |
| ZDHHC9 | 2.06E-06 | -4.66844 | 0.021829 | -2.52794 |
| ZNF395 | 0.000505 | -1.53281 | 0.017217 | -2.13114 |
| ZNF828 | 1.17E-05 | -2.19915 | 0.008483 | -2.37284 |
| ZYX | 0.000562 | -1.78902 | 0.033837 | -1.79776 |
